# A multi-lab experimental assessment reveals that replicability can be improved by using empirical estimates of genotype-by-lab interaction

**DOI:** 10.1371/journal.pbio.3002082

**Published:** 2023-05-01

**Authors:** Iman Jaljuli, Neri Kafkafi, Eliezer Giladi, Ilan Golani, Illana Gozes, Elissa J. Chesler, Molly A. Bogue, Yoav Benjamini

**Affiliations:** 1 Department of Statistics and Operations Research, Tel-Aviv University, Tel-Aviv, Israel; 2 Department of Epidemiology and Biostatistics, Memorial Sloan Kettering Cancer Center, New York, New York, United States of America; 3 School of Zoology, Faculty of Life Sciences, Tel Aviv University, Tel Aviv, Israel; 4 The Elton Laboratory for Molecular Neuroendocrinology, Department of Human Molecular Genetics and Biochemistry, Sackler Faculty of Medicine, Sagol School of Neuroscience and Adams Super Center for Brain Studies, Tel Aviv University, Tel Aviv, Israel; 5 The Sagol School of Neuroscience, Tel Aviv University, Tel Aviv, Israel; 6 The Jackson Laboratory, Bar Harbor, Maine, United States of America; University of Edinburgh, UNITED KINGDOM

## Abstract

The utility of mouse and rat studies critically depends on their replicability in other laboratories. A widely advocated approach to improving replicability is through the rigorous control of predefined animal or experimental conditions, known as standardization. However, this approach limits the generalizability of the findings to only to the standardized conditions and is a potential cause rather than solution to what has been called a replicability crisis. Alternative strategies include estimating the heterogeneity of effects across laboratories, either through designs that vary testing conditions, or by direct statistical analysis of laboratory variation. We previously evaluated our statistical approach for estimating the interlaboratory replicability of a single laboratory discovery. Those results, however, were from a well-coordinated, multi-lab phenotyping study and did not extend to the more realistic setting in which laboratories are operating independently of each other. Here, we sought to test our statistical approach as a realistic prospective experiment, in mice, using 152 results from 5 independent published studies deposited in the Mouse Phenome Database (MPD). In independent replication experiments at 3 laboratories, we found that 53 of the results were replicable, so the other 99 were considered non-replicable. Of the 99 non-replicable results, 59 were statistically significant (at 0.05) in their original single-lab analysis, putting the probability that a single-lab statistical discovery was made even though it is non-replicable, at 59.6%. We then introduced the dimensionless “Genotype-by-Laboratory” (GxL) factor—the ratio between the standard deviations of the GxL interaction and the standard deviation within groups. Using the GxL factor reduced the number of single-lab statistical discoveries and alongside reduced the probability of a non-replicable result to be discovered in the single lab to 12.1%. Such reduction naturally leads to reduced power to make replicable discoveries, but this reduction was small (from 87% to 66%), indicating the small price paid for the large improvement in replicability. Tools and data needed for the above GxL adjustment are publicly available at the MPD and will become increasingly useful as the range of assays and testing conditions in this resource increases.

## Abstract 2

The random laboratory model allows investigators to estimate whether an experimental result is likely to replicate based on the typical noise that exists among laboratories that use similar endpoints. This study uses new experimental data to show that such a model, when applied to archived data, improves prediction of replicability for experiments.

## Introduction

The scientific community is concerned with issues of published results that fail to replicate in many fields including those of preclinical animal models, drug discovery, and discovering mammalian gene function [1–3]. Many reports have called out a “crisis” in replicability as an explanation for translational failures for preclinical models. Indeed, some of the first concerns regarding the complex interaction between genotype and the conducting laboratory were raised in the field of rodent behavioral phenotyping [4]. While mouse and rat models may predict the human situation, such as the case of activity-dependent neuroprotective protein (ADNP) and the potential of its fragment as a drug (reviewed in Gozes [5]), the utility of any findings critically depends on their replicability in other laboratories [6–8]. A similar concern arises regarding the interaction between the conducting laboratory and novel pharmacological treatments (e.g., Rossello and colleagues [9]) that are of vital importance for translational research into novel drug development.

It should be emphasized that the impact of such animal studies goes well beyond animal behavior to clinical studies in neurology and psychiatry. These clinical studies, requiring multiple research centers, are much less homogeneous in terms of genetic and environmental backgrounds of the treatment cohorts. As such, many failures are noted in clinical studies employing therapies deemed efficacious in animal studies. As Collins and Tabak wrote when discussing these problems in preclinical animal studies “If the antecedent work is questionable and the trial is particularly important, key preclinical studies may first need to be validated independently” [10].

In response, there have been several attempts to refine experimental design and practice, in an attempt to extract a pure treatment effect. In most cases, a radical push toward standardization of laboratory conditions, genotypes, and other study conditions has been advocated. However, such attempts are misguided, as effects are often dependent on idiosyncratic conditions, and therefore, standardization produces exactly the opposite of the intended effect—rather than increase replicability; it limits generalizability to the narrow range of conditions under which the finding was obtained. This is sometimes referred to as the “the standardization fallacy” [11,12]. The problem intensifies if the usual recommendation to increase power by larger sample size is followed, for now there is high power to find even small effects particular to the study. One should instead seek to estimate the extent to which a discovery is replicable across the range of likely conditions. For this purpose, heterogenization or systematic variation of testing conditions have been advanced as a strategy; however, both approaches increase experimental costs through somewhat larger sample sizes [7,12]. Moreover, these efforts are yet to prove practical and useful [8,13].

In a previous publication [14], we proposed an alternative to standardization or heterogenization in order to assess statistically the replicability of single-lab results, before making the effort to replicate them across multiple labs. The statistical approach hinges on the “Random Lab Model” for the measured phenotype of a specific genotype in a particular [15]. In particular, we considered a result to be “replicable” if it is tested in a multi-lab experiment and was statistically significant under the assumptions of the random lab model (0.05 level is used throughout the paper). This model treats both the effect of the lab, and more importantly, the effect of the interaction of this genotype in this particular lab, as random. The random effect of the lab cancels out when comparing 2 genotypes in the same lab, but the random interaction contributions add up. Moreover, the actual interaction effect cannot be separated from the lab effect in the analysis of the single-lab results. Still, it can be separated in multi-lab experiments, and while the values are irrelevant to a new lab, their standard deviation is relevant and can be estimated.

We therefore suggested to estimate the interlaboratory replicability of novel discoveries in a single-lab study in the following way: We first estimate the Genotype by Laboratory (GxL) interaction standard deviation in previous data from other labs and possibly other genotypes. We then adjust the within-groups standard deviation, which is usually used for testing confidence intervals in a single-lab analysis, by inflating it with the GxL interaction standard deviation (see Statistical methods). This “GxL adjustment” thus generates a larger yardstick, against which genotype differences are tested, and confidence intervals are reported. Consequently, this adjustment raises the benchmark for discovering a significant genotype effect, trading some statistical power for better replicability. We demonstrated that previous phenotyping results from multi-lab databases can be used to derive a GxL-adjustment term to ensure (within the usual 0.05 error) the replicability of single-lab results, for the same phenotypes and genotypes, even before making the effort of replicating the findings in additional laboratories [14].

This demonstration, however, still raises several important questions. Kafkafi and colleagues used data from a highly coordinated [14], multi-lab phenotyping program to estimate the standard deviations of the GxL interaction for each phenotype. These were then used to adjust the results of each of these same labs separately. While the success of this demonstration is encouraging, it does not cover the more realistic setting where the adjusted laboratories are operating independently from the laboratories used for generating the GxL adjustment. Here, we investigate the question of whether GxL adjustment of single-lab results from independently collected data in other labs, reduces the proportion of single-lab discoveries among the non-replicable discoveries, relative to the naïve analysis, and what loss of power does it involve.

A related important question is whether GxL estimation from standardized studies can be used to successfully identify replicable results in studies that were not subject to the same standardization. Namely, will the adjustment based on the data from the International Mouse Phenotyping Consortium (IMPC) [16,17], which typically uses relatively well-coordinated, standardized protocols, predict the replicability of results obtained in more common and realistic scenarios, such as those deposited by many investigators into the Mouse Phenome Database (MPD Phenome.jax.org [18]). Unlike the IMPC, MPD archives previously conducted studies, which were not a priori meant to be part of a multi-lab project. Their methods, apparatus, endpoints, and protocols of such experiments are thus not expected to be standardized.

Finally, our previous demonstration of GxL adjustment tested only genotype effects, using inbred strains and knockouts, but not pharmacological effects. It therefore remains to be tested whether the pre-estimated interaction of treatment with lab (TxL) or the interaction of the genotype and pharmacological treatment with the lab (GxTxL) can also be used to adjust single-lab treatment testing in a similar way.

In order to enable such studies, we modify our previous GxL-adjustment by introducing the dimensionless *GxL-factor* per phenotype and subpopulation, being the ratio of the interaction standard deviation to the pooled within groups standard deviations. The intuition underlying this factor can be explained by the simplistic situation where one lab measures distance traveled in inches, while in multiple benchmarking labs (the multi-lab) it is measured in centimeters. Standard deviations are affected by the unit of measurement, so one cannot transfer the interaction standard deviation from the centimeter-based multi-lab experiment as a proxy for the interaction standard deviation in the inches-using lab. However, taking the ratio of the interaction standard deviation to the pooled measured standard deviations from the multi-lab analysis defines a scale-free factor that will be the same in the single lab. Now, taking the GxL-factor from the multi-lab and multiplying it back by the standard deviation within groups in the new lab will produce the right value (in inches). Turning to a more realistic situation where a widely used activity measure, “percent time spent at center” is measured by 2 different systems with some variation in the definition of “center,” we still expect that the GxL-factor will be quite stable across labs (also termed “environmental effect ratio” by Higgins and colleagues [19]). Thus, the use of the scale-free dimensionless GxL-factor enables us to carry the information about the interaction of a phenotype to other laboratories, other genotypes, and variations in setups and conditions.

In the present study, we assessed the value of the GxL-adjustment for experimental results previously submitted to the MPD, involving genotype effects on several phenotypes, as well as fluoxetine treatment effect on various genotypes. For this purpose, we conducted an experiment measuring the above phenotypes on several genotypes across 3 labs, without strong interlaboratory standardization and coordination. The replications obtained in our own experiment enabled us to estimate the GXL parameter to identify the non-replicable discoveries from MPD. Counting how many of these were statistically significant in their original study, this proportion is an estimate of the probability that a statistical discovery is made even though it is not replicable. A convenient terminology for this probability is the “Type-I replicability error,” in analogy to the Type-I error in testing, being the probability of making a statistical discovery even if there is no effect. We could thereby show that using the GxL adjustments in the original studies would have greatly reduced the number of non-replicable discoveries, and thereby reduce this Type-I replicability error. We therefore recommend supplementing any single-lab discovery with a GxL-adjusted analysis as an assessment of whether it is predicted to be replicated across multiple labs.

## Results

We conducted the phenotyping experiment (“3-lab experiment”) in the following 3 laboratories: the Center for Biometric Analysis core facility in The Jackson Laboratory, USA (JAX); the George S. Wise Faculty of Life Sciences, Tel Aviv University, Israel (TAUL); and in the Faculty of Medicine, Tel Aviv University, Israel (TAUM). We compare the effects of 6 mouse genotypes and 1 pharmacological treatment (18 mg/kg fluoxetine), on several behavioral phenotypes, as well as on 1 readily obtainable physiological phenotype (body weight).

These were chosen to replicate some of the original results as reported in previous studies submitted to MPD: Wiltshire2 (Benton and colleagues [20]: Open-Field (OF), Tail-Suspension (TS); Tarantino2 [21]: OF; Crabbe4 [22]: Grip Strength (GS); Tordoff3 [23]: Body Weight (BW); Crowley1 [24]: BW. The study code names are those used in the MPD website. These 152 comparisons were chosen to reflect comparisons we could efficiently evaluate in the 3-lab experiment (see Methods and S1, S2 and S5 Tables in Supporting information).

**Fig 1:**
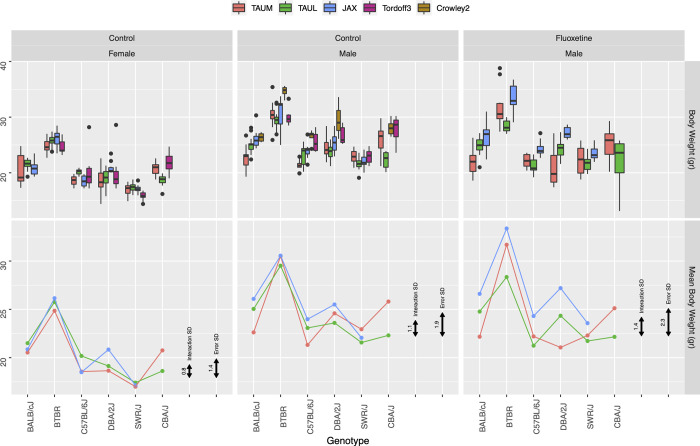
BW in the 3 labs and in previously published studies in MPD Crowley1 and Tordoff3, using boxplots (top) and genotype means (bottom) in the 3 laboratories, in females (left), males (center), and fluoxetine-treated males (right). Each boxplot (top) displays the results for the corresponding genotype on the horizontal axis, in 1 lab identified by color, where 3 boxplots correspond to the 3 labs. When available for a given genotype, a fourth boxplot displays the corresponding result from the original MPD study, in this case the Crowley1 study which was performed in females or the Tordoff3 study which was performed in males. Black doubly arrowed bars represent the standard deviation of the Genotype-by-Lab interaction (left), and the within-group standard deviation (right). The estimated GxL factor is the ratio of the length of the left double arrowed bar to the right one. The data and R code underlying this figure can be found in https://doi.org/10.5281/zenodo.7672211. BW, body weight; MPD, Mouse Phenome Database.

**Fig 2:**
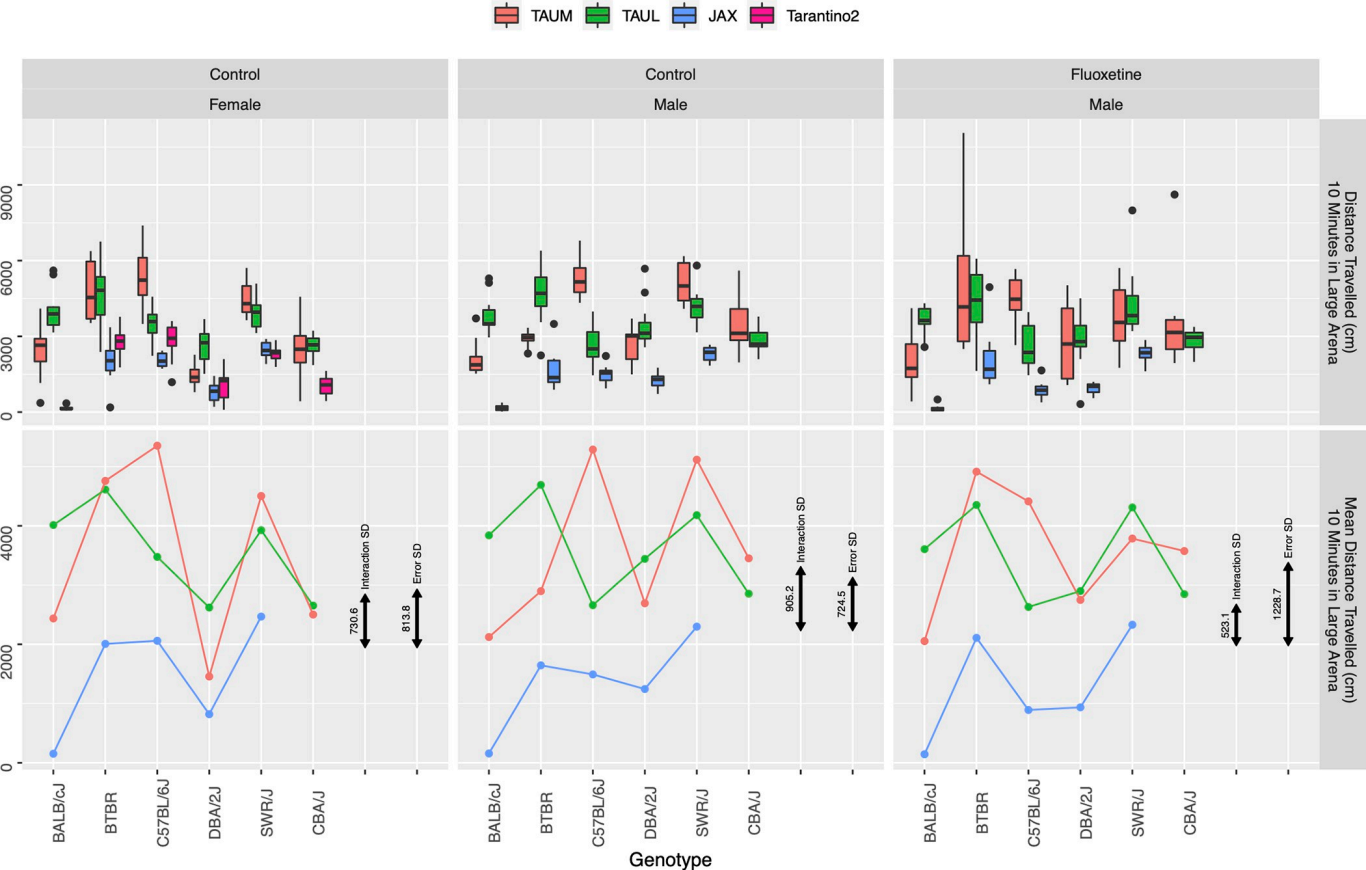
DT in the 3-lab experiment, in a large arena for 10 min, and the MPD study Tarantino2 (for females). Graph organization is as in Fig 1, using boxplots (top) and genotype means (bottom) in the 3 laboratories: in females (left), males (center), and fluoxetine-treated males (right). Black doubly arrowed bars represent the standard deviation of the Genotype-by-Lab interaction (left) and the within-group standard deviation (right). The estimated GxL factor is the ratio of the left double arrowed bar to the right one. The data and R code underlying this figure can be found in https://doi.org/10.5281/zenodo.7672211. DT, distance traveled; MPD, Mouse Phenome Database.

Most of these phenotypes were also measured in multiple labs in the IMPC database, which served as a second source for estimating the GxL-factors, analyzed in Kafkafi and colleagues [14]. All phenotyping results used in the experiment are presented in Figs 1, 2 and S1–S5. The research process in the following Results section is summarized by the flowchart in Fig 5.

### Assessing replication of the MPD results using the 3-lab experiment

We first use our 3-lab experiment to evaluate whether the chosen 152 results reported in the MPD deposited studies are replicable or not, using the Random Lab Mixed Model analysis. Fig 1 displays the 3-lab results and their summaries for body weight, a commonly used physiological measure. It also displays the standard deviation of the within-group error and the standard deviation of the GxL interaction. Fig 2 does the same for the commonly used behavioral measure of distance traveled. S1–S5 Figs display the phenotyping results for the other phenotypes.

Fig 1 demonstrates the concepts underlying the Random Lab Model. There are some consistent additive differences between labs, expressed as vertical distances between the lines for labs in Fig 1 (and in Figs 2 and S1–S5), but these will not affect the replicability when comparing genotypes within the same lab [6]. The concern is rather the genotype-by-lab (GxL) interaction, which can be perceived by differences in slope of the lines connecting one genotype to the next, across labs (e.g., C57BL/J6, DBA/2J, and SWR/J genotypes for fluoxetine treated), while parallel slopes represent no GxL interaction (e.g., BALB/cJ, BTBR, and C57BL/J6 genotypes for females). The Random Lab Model treats these slope variations as random, and takes them into consideration when using the 3-lab experiment to decide whether the original discovery was replicable. Testing the 152 comparisons by the Random Lab Model, we found that 53 of the results were replicable, so the other 99 were considered non-replicable. Adhering to this definition of “replicable discoveries,” throughout this paper, avoids terminologies such as “true discoveries” or “ground truth,” which is beyond the evidence we have. It is important to realize that both definitions are based on statistical tests, and inherit the uncertainties involved, so that a “non-replicable discovery” may still replicate in some future study.

### The GxL-adjustment factor

In this section, we use the results of our 3-lab experiment as a surrogate for a database, which has results on phenotypes measured for multiple genotypes in several nonstandardized labs, to extract the GxL adjustment factor. For each phenotype and for the 3 subpopulations of animals (untreated males, untreated females, and fluoxetine-treated males), the within-group standard deviation *σ* summarizes the variability displayed by the boxplots (this standard deviation is represented in the figures by the bottom-right black arrow). The interaction between Genotype and Lab is summarized by the standard deviation *σ*_*G*×*L*_ (represented by the bottom-left black arrowed bar). The ratio of the latter to the first standard deviations is the estimated GxL-factor, which is dimensionless and noted by *γ*. We utilize this factor to inflate the usual standard error used in single-lab analysis for *t* tests and confidence intervals: The larger this factor is, the longer the confidence intervals are, and the higher the *p*-values are (see Statistical methods 4.5.1).

As examples illustrating the calculation of the GxL-factor, consider first the reliably measured and well-defined physiological phenotype body weight (Fig 1). The standard deviation of the error within the groups is small relative to the average weight, about 1.4/20 = 0.07 for females (left panel). Similarly, the size of the interaction between genotype and lab 0.8/20 = 0.04 is also small. Yet the GxL-factor, the ratio of these 2 standard deviations, is not negligible: *γ* = 0.8/1.4 = 0.57. In the fluoxetine-treated males (right panel), the GxL-factor remains about the same *γ* = 0.61, although both the interaction standard deviation and the standard deviation of the results within groups increased, in fact by more than 50% (as evident from the arrowed bars).

In contrast, for the distance traveled (DT) in 10 min session endpoint, the estimated value of ***γ*** was near or larger than 1 (note especially Fig 2 middle, where the Interaction SD bar is larger than the Error SD bar, so *γ*>1).

### GxL-adjustment of phenotyping results

#### GxL-adjustment of independent labs

**Fig 3:**
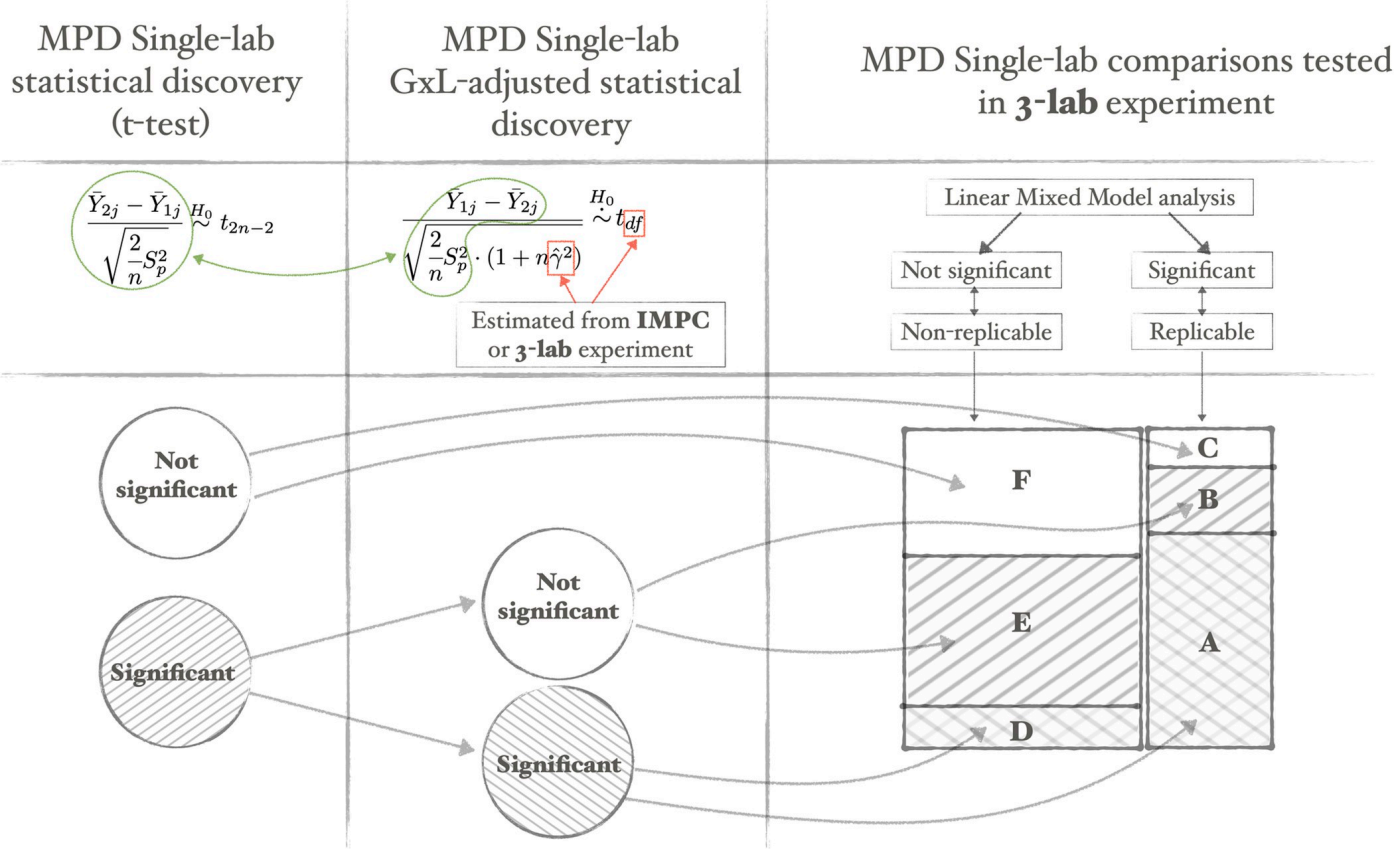
The GxL-adjustment process and the analysis of its properties in terms of type I replicability error and replicability power. The 45° shading is for the original significant single-lab discoveries, containing A, B, D, and E; the 135° shading is for the significant GxL-adjusted discoveries, containing A and D, but since any such discovery will also be an original single-lab discovery the shading appears crossed. The type I replicability error is the area of D relative to the left column. The power is the area of A relative to the right column. The area of E reflects the reduction in non-replicable discoveries over the original single-lab discoveries; the area of B reflects the loss of power. The visualization of the results in the bottom right display is approximately based on the data of 152 comparisons reported in Table 1, and the categories A–F correspond to those appearing in Tables 1–4.

We now reanalyze the original statistically significant discoveries reported in MPD database, using the GxL-adjustment factors described in 2.2, and incorporating them into the original *t* tests (see Fig 3 and Statistical methods).

**Table 1:** Results of naïve and GxL-adjusted genotypic differences for all phenotypes in the MPD experiments, using GxL-factors estimated from the 3-lab.

Assessed by 3-lab study	Single-lab difference	Single-lab GxL-adjusted	Category	Interpretation		No. cases
Replicable	significant	significant	A	Replicable single-lab discoveries, approved by adjustment		35
Replicable	significant	ns	B	Replicable single-lab discoveries, missed by adjustment		11
Replicable	ns	ns	C	Replicable discoveries, missed by both in the single lab		7
Non-replicable	significant	significant	D	Non-replicable single-lab discoveries, insufficient adjustment		12
Non-replicable	significant	ns	E	Non- replicable single-lab discoveries, prevented by adjustment		47
Non-replicable	ns	ns	F	Non-replicable discoveries prevented by both in the single lab		40
**Category**	**Interpretation**			**No. cases**	**% Cases**	**(95% CI)**
(D+E)/(D+E+F)	**Estimated type I replicability error of a single-lab discovery**			**59/99**	**59.6%**	(49–69)
(D)/(D+E+F)	**Estimated type I replicability error of GxL-adjusted single-lab discovery**			**12/99**	**12.1%**	(6–20)
(A+B)/(A+B+C)	Estimated power of a single-lab discovery			46/53	86.7%	(77–94)
(A)/(A+B+C)	Estimated power of GxL-adjusted single-lab discovery			35/53	66.0%	(53–79)

(a) Raw results, (b) proportions; the first 2 proportions serve as estimates of “type I replicability error,” where the error is making a single-lab discovery while it is non-replicable, and the last 2 serve as estimates of power to make a single-lab statistical discovery when the result is replicable.

**Table 2:** Results of naïve and GxL-adjusted genotypic differences for all phenotypes in the MPD experiments, using GxL-factors estimated from the IMPC standardized data.

Assessed by 3-lab study	Single-lab difference	Single-lab GxL-adjusted	Category	Interpretation		No. cases
Replicable	significant	significant	A	Replicable single-lab discoveries, approved by adjustment		29
Replicable	significant	ns	B	Replicable single-lab discoveries, missed by adjustment		1
Replicable	ns	ns	C	Replicable discoveries, missed by both in the single lab		3
Non-replicable	significant	significant	D	Non-replicable single-lab discoveries, insufficient adjustment		14
Non-replicable	significant	ns	E	Non- replicable single-lab discoveries, prevented by adjustment		16
Non-replicable	ns	ns	F	Non-replicable discoveries prevented by both in the single lab		29
**Category**	**Interpretation**			**No. cases**	**% Cases**	**(95% CI)**
(D+E)/(D+E+F)	**Estimated type I replicability error of a single-lab discovery**			**30/59**	**50.8%**	(37–63)
(D)/(D+E+F)	**Estimated type I replicability error of GxL-adjusted single-lab discovery**			**14/59**	**23.7%**	(13–36)
(A+B)/(A+B+C)	Estimated power of a single-lab discovery			30/33	90.9%	(79–100)
(A)/(A+B+C)	Estimated power of GxL-adjusted single-lab discovery			29/33	87.8%	(76–97)

(a) Raw results, (b) proportions; the first 2 proportions serve as estimates of “type I replicability error,” where the error is making a single-lab discovery while it is non-replicable, and the last 2 as estimates of power to make a statistical discovery when the result is replicable.

**Table 3:** Results of naïve and GxLxT-adjusted genotypic differences for Wiltshire2 experiment, using GxLxT factors estimated from the 3-lab data.

								Fluoxetine effect in genotypes	Genotypic differences in fluoxetine effect	
Assessed by 3-lab study	Single-lab difference	Single-lab GxL-adjusted	Category	Interpretation				No. cases	No. cases	
Replicable	significant	significant	A	Replicable single-lab discoveries, approved by adjustment				1	0	
Replicable	significant	ns	B	Replicable single-lab discoveries, missed by adjustment				1	1	
Replicable	ns	ns	C	Replicable discoveries, missed by both in the single lab				2	8	
Non-replicable	significant	significant	D	Non-replicable single-lab discoveries, insufficient adjustment				1	0	
Non-replicable	significant	ns	E	Non- replicable single-lab discoveries, prevented by adjustment				2	1	
Non-replicable	ns	ns	F	Non-replicable discoveries prevented by both in the single lab				11	35	
					**Fluoxetine effect in genotypes**			**Genotypic differences in fluoxetine effect**		
**Category**	**Interpretation**				**No. cases**	**% Cases**	**(95% CI)**	**No. cases**	**% Cases**	**(95% CI)**
(D+E)/(D+E+F)	**Estimated type I replicability error of a single-lab discovery**				3/14	21.4%	(0–43)	1/36	2.8%	(0–8)
(D)/(D+E+F)	**Estimated type I replicability error of GxL-adjusted single-lab discovery**				1/14	7.1%	(0–21)	0/36	0.0%	)0–10)
(A+B)/(A+B+C)	Estimated power of a single-lab discovery				2/4	50%	(0–100)	1/9	11.1%	(0–33)
(A)/(A+B+C)	Estimated power of GxL-adjusted single-lab discovery				1/4	25%	(0–75)	0/9	0.0%	(0–34)

The rightmost 2 columns present results regarding differences in fluoxetine effect between genotypes, and the 2 columns left of them represent results regarding fluoxetine effect in single genotypes.

**Table 4:** Results of naïve testing and lower significance level of 0.005 genotypic differences for all phenotypes in the MPD experiments.

Assessed by 3-lab study	Single-lab difference	Single-lab GxL-adjusted	Category	Interpretation		No. cases
Replicable	significant	significant	A	Replicable single-lab discoveries, approved by adjustment		38
Replicable	significant	ns	B	Replicable single-lab discoveries, missed by adjustment		8
Replicable	ns	ns	C	Replicable discoveries, missed by both in the single lab		7
Non-replicable	significant	significant	D	Non-replicable single-lab discoveries, insufficient adjustment		24
Non-replicable	significant	ns	E	Non- replicable single-lab discoveries, prevented by adjustment		35
Non-replicable	ns	ns	F	Non-replicable discoveries prevented by both in the single lab		40
**Category**	**Interpretation**			**No. cases**	**% Cases**	**(95% CI)**
(D+E)/(D+E+F)	**Estimated type I replicability error of a single-lab discovery**			**30/59**	**50.8%**	(37–63)
(D)/(D+E+F)	**Estimated type I replicability error of GxL-adjusted single-lab discovery**			**14/59**	**23.7%**	(13–36)
(A+B)/(A+B+C)	Estimated power of a single-lab discovery			30/33	90.9%	(79–100)
(A)/(A+B+C)	Estimated power of GxL-adjusted single-lab discovery			29/33	87.8%	(76–97)

Note that a nonsignificant original single-lab result cannot become significant after the adjustment, so we reanalyze the original statistically significant discoveries only, and examine the implications of the adjustment on the estimated probability that that a single-lab statistical discovery was made even though it is non-replicable (see “Statistical methods” and Table 5). In analogy to the type I error in testing being the probability of making a statistical discovery even if there is no effect, a coin a convenient terminology for this probability, namely type I replicability error. Table 1 presents the results.

**Table 5:** The results tables in the “Results” section use the same structure, categories, and interpretation.

Assessed by 3-lab study	Single-lab difference	Single-lab GxL-adjusted	Category	Interpretation
Replicable	significant	significant	A	Replicable discoveries, approved by adjustment
Replicable	significant	ns	B	Replicable discoveries, missed by adjustment
Replicable	ns	ns	C	Replicable discoveries, missed by both in the single lab
Non-replicable	significant	significant	D	Non-replicable single-lab discoveries, insufficient adjustment
Non-replicable	significant	ns	E	Non- replicable single-lab discoveries, prevented by adjustment
Non-replicable	ns	ns	F	Non-replicable discoveries prevented by both in the single lab
			(D+E)/(D+E+F)	Estimated type I replicability error of a single-lab discovery
			(D)/(D+E+F)	Estimated type I replicability error of GxL-adjusted single-lab discovery
			(A+B)/(A+B+C)	Estimated power of a single-lab discovery
			(A)/(A+B+C)	Estimated power of GxL-adjusted single lab

Out of the 99 non-replicable results in our 3-lab study, 59 were statistically significant (at 0.05) in their original single lab analysis, estimating the type I replicability error of the original analysis at 60% (59/99), see S6 Table in the Supporting information for a list of the 53 replicable discoveries. GxL-adjustment considerably decreased this proportion to 12% (12/99). The price paid in decreased power to detect replicable discoveries was a decrease from 87% (46/53) to 66% (35/53). In absolute terms, 47 non-replicable “discoveries” were prevented, while only 11 replicable discoveries were missed.

#### GxL-adjustment using IMPC data

**Fig 4:**
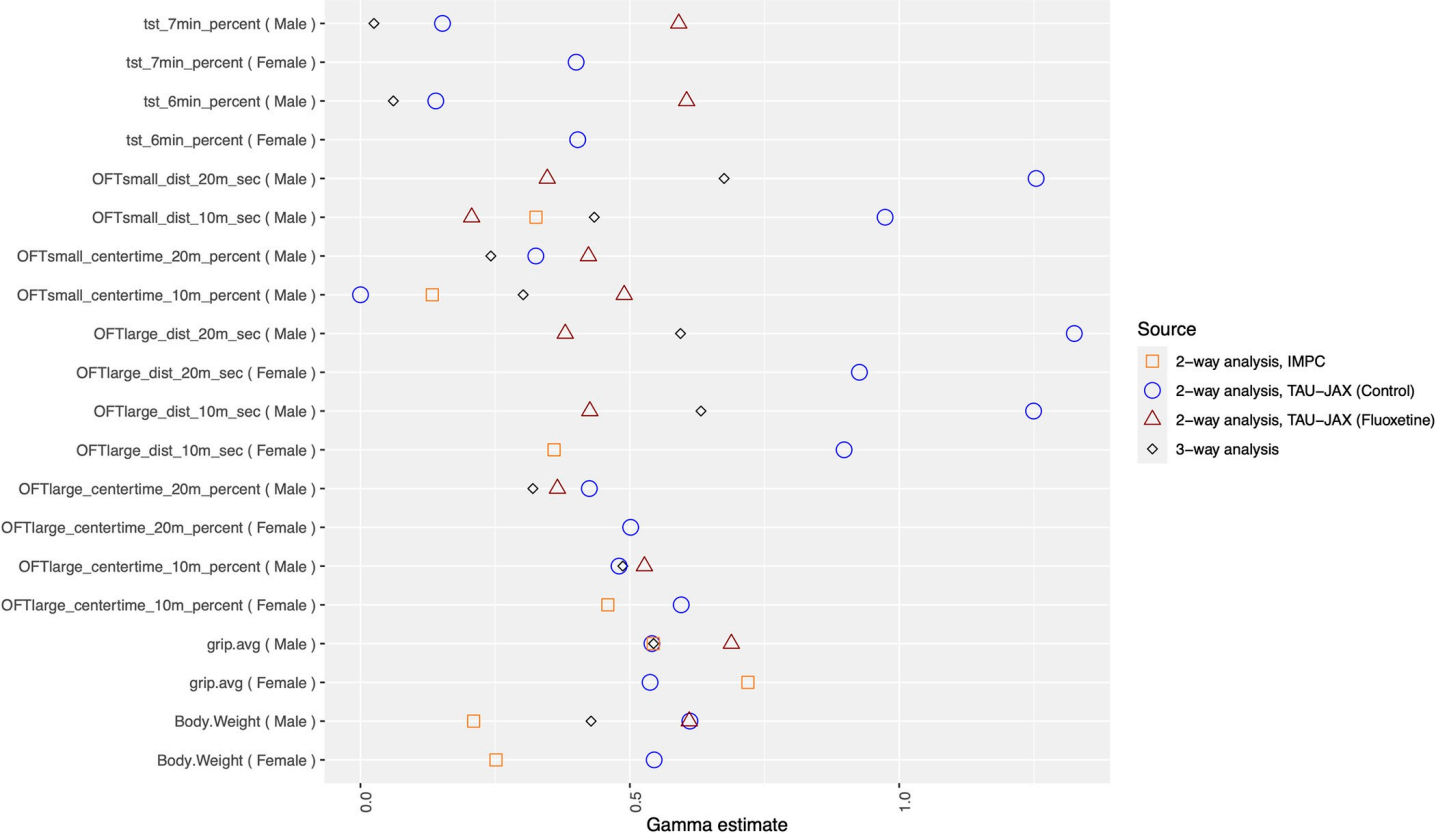
The values of the estimated interaction factor *γ*, for all measures, as estimated from various sources: GxL-factor from our 3-labs control data and from our fluoxetine treated data; GxL-factor from IMPC data; TxGxL-factor from our 3-labs data. CT and TS were logit transformed and GS was raised to the power of 1/3, to bring the distributions close to Gaussian. The transformations were used for these phenotypes throughout the analysis and the adjustment. The data and R code underlying this figure can be found in https://doi.org/10.5281/zenodo.7672211. CT, center time; GS, grip strength; TS, tail suspension.

We now investigate whether GxL estimation from IMPC database, which typically uses relatively well-coordinated, standardized protocols, can be used to adjust single-lab experiments that do not strictly adhere to these protocols. We therefore use the GxL-interactions previously calculated from the IMPC multi-lab database [14]. The values of *γ* for the different endpoints are presented in S3 Table and displayed in Fig 4. Since IMPC database does not contain data of fluoxetine-treated mice, and does not include a TS test, the number of differences available for the study is only 92. The number of non-replicable discoveries in this smaller pool is 59 phenotypic differences. Table 2 presents the results.

Using the IMPC-derived GxL-adjustment decreased the proportion of single-lab MPD statistical discoveries among the non-replicable discoveries from 51% to 24%, for the price in decreasing the power to detect replicable discoveries from 91% with no adjustment to 87%. In absolute terms, 16 non-replicable “discoveries” were prevented, while only 1 replicable discovery was missed in the combined dataset.

In order to compare the results of adjustment based on the coordinated experiments in the IMPC with results using the more heterogeneous MPD-based GxL-adjustment, we have limited the 152 comparisons previously analyzed in 2.3.1 to the 92 differences that can be analyzed both by MPD and IMPC GxL factors. Instead of 24% proportion of GxL-adjusted single-lab discoveries among the non-replicables, for IMPC-based GxL-adjustment, it is 10% when the adjustment is based on the 3-lab GxL-factors. The power in the IMPC-based adjustment is 91%, in comparison to 61% using 3-lab-based adjustment (see S4 Table).

### Using GxL-adjustment for comparing drug effects across genotypes

#### Fluoxetine effect across 6 shared genotypes

Our 3-lab experiment shared 6 genotypes and 3 phenotypes with the Wiltshire2 study in the MPD, which estimated the effect of fluoxetine treatment. This study offered 15 treatment effects in genotypes and 45 pairwise comparisons of treatment effects between genotypes, where the “ground truth” could be derived from our 3-lab data, using a three-way random lab analysis with genotype-by-lab-by-treatment interaction. This interaction term is also relevant for adjusting single-lab comparisons of fluoxetine treatment between genotypes, so it could be estimated from the 3-lab experiment (see Statistical methods.)

The number of results available for fluoxetine effect in genotypes is small, so the results of their adjustment should be viewed with much caution. The number of results for pairwise comparisons of fluoxetine effects between genotypes is larger, but most of them do not result in significant difference in the original study. Combining the 2 sets of results, the number of non-replicable discoveries is 50. Thus, the estimated type I replicability error was reduced from 8% (4/(14+36)) to 2% (1/(14+36)) by the adjustment, and the power to detect replicable discoveries was reduced from 23% (3/13) to 8% (1/13).

#### Fluoxetine effect across 30 genotypes

The Wiltshire2 MPD study included 30 genotypes, between which 1,305 pairwise comparisons of treatment effect can be conducted, with 45 of these included in our 3-lab experiment (already reported in the previous section and Table 3). These 1,305 comparisons can be used to assess the effect of the GxL-adjustment using GxL-factor as estimated from the 3-lab experiment for the 3 phenotypes. However, we still cannot assess the replicability of these differences, since we did not have these genotypes in our experiment.

For 276 comparisons, the linear contrasts for fluoxetine treatment effects were found significant in Wiltshire2 data (using t-distribution). Of these, 204 (73%, 95% CI 68.5 to 79.0) would become nonsignificant once the GxLxT-adjustment is used. The adjustment thus weeds out a large proportion of apparently significant differences, but we cannot tell if it is justifiable or not.

### Lowering the significance threshold in the original analysis

One potential response to our previous results is that by incorporating the GxL-adjustment we have merely lowered the alpha level of the test being used, and one can instead use a single lower threshold for significance across all comparisons. We therefore have tested the universal recommendation by Benjamin and colleagues to use the 0.005 level for statistical significance in the original studies [25]. Table 4 shows the rejection rates when adapting the uniformly more conservative threshold.

Using the lower *α* = 0.005 than indeed offers more conservative protection, where the type I replicability error is reduced to 24%, compared to the 60% using the usual *α* = 0.05 test in the single lab. However, this still double the 12% type I replicability error obtained using GxL-adjustment. The power is 72%, somewhat higher than the 66% power obtained using the GxL-adjustment.

### Comparing GxL-factors across phenotypes and subpopulations

The within-group standard deviation, which is divided by the square root of the sample size to get the standard error, decreases with increased number of animals per group. In contrast, the impact of the GxL-factor does not diminish with increased number of animals per group, and hence, the importance of its magnitude. We use the results to take a comparative look at the magnitude of the factor across subpopulation, database being used and phenotypes. In our experiment, we could also measure related phenotypes and variations in setups (such as 20 min session duration in OF, instead of 10 min) that were not required for adjusting an MPD experiment. We still estimated *γ* for them. These are all presented in Fig 4 and in S3 Table.

- In some behavioral phenotypes, notably the percent time spent immobile in the tail suspension (TS) test (S4 Fig), there were large absolute differences between labs. This is hardly surprising, considering our use of different measurement technologies (force transducer method in JAX, as opposed to video tracking in TAUM and TAUL), as well as the choice of analysis parameters, such as the cutoff value for detecting immobility, which was also left for the specific lab to determine, as in typical single-lab studies. Despite this, the standard deviation of the GxL interaction is still considerably smaller than that of the error within labs, and the GxL-factor is small.
- As noted before, for the DT endpoint in subpopulations, the estimated value of *γ* was near 1 for 10 min session duration and larger than 1 for 20 min duration. They were similar for male and female subpopulations. These high *γ* values do not appear to be the fault of any single genotype, lab, sex, or treatment. Indeed, large *γ* occurred in all endpoints of DT, while for the (logit transformed) Center Time (CT) endpoints, which were measured in the same open field (OF) sessions were about half the size, and in the range of the other endpoints (see Discussion).
- Comparing the adjustment offered by *γ*, as estimated from the standardized IMPC data to the adjustment offered by *γ* estimated from our nonstandardized 3-lab study, we notice that the latter tend to be larger (see Fig 4). This is expected, as explained in the Discussion (3.2). Indeed, using IMPC-based GxL-factor estimates resulted in 24% of non-replicable adjusted original discoveries versus the 10% when using the 3-labs experiment to estimate them.
- The interaction estimated from the 3-way analysis of treatment-by-genotype-by-lab tends to be smaller than the GxL-factor in the fluoxetine treated and this tends to be smaller than the GxL-factor of the untreated. This may indicate that using the GxL-factor with a new treatment might serve as an upper bound for the unknown treatment-by-lab TxL factor.

## Discussion

### The contribution of GxL-adjustment to replicability

What performance can be expected from GxL-adjustment in realistic situations of single-lab studies, which are often not standardized with other labs? The most direct answer to this question is given in Section 2.2.1. Using database-derived interaction estimates from a multi-lab study for body weight (BW), distance traveled (DT), and center time (CT) in the open field test, forelimb grip strength (GS), and percent time immobile in TS, the GxL-adjustment reduced the probability of discovering a non-replicable result from 60% to 12%. This 48 percent points reduction came with a reduction of power, from 87% to 66%, relatively small compared to great reduction in failures to replicate. In absolute terms, in the combined mega-experiment used for our study 47 non-replicable “discoveries” were prevented, while only 11 replicable discoveries were missed. It is important to emphasize that the original studies used only a few of the genotypes from which GxL-interactions were estimated. The testing parameters and conditions of testing were also somewhat different in both.

It might be argued that our success is merely due to our more stringent thresholds for significance. Lowering the significance threshold to 0.005, as suggested in Benjamin and colleagues [25], and the many supporters that signed on this suggestion at the time of its publication, will have similar results. However, as shown in Section 2.4, when implementing this suggestion for the above set of MPD original results, the result was more conservative than the original analysis, but the type I replicability error was 24%, double that for the GxL adjustment, with the power in between. Thus, we observed that for a small sacrifice in power, a much greater improvement in replicability can be obtained. The GxL-adjustment takes into consideration the different levels of adjustment needed for different endpoints when facing the multi-lab replicability challenge, while using a single lower statistical discovery threshold across all phenotypes ignores the nature and robustness of each individual phenotype. Clearly, there is an advantage to offering differing yardsticks to different phenotypes.

It should be emphasized that in order to isolate the effect of GxL-adjustment on replicability, we have treated each original result as though it were individually generated, ignoring any selection effect of the statistically significant ones among the many ones tested. Analyzing the structure of each original experiment and controlling the false discovery rate in the original studies would have further reduced the number of non-replicated results below 12%, but this is beyond the goals of the current study, which are to address the biological variability. Similarly, when using the GxL adjustment for *p*-values and confidence intervals, concern about multiple comparisons should not be neglected, for otherwise the non-replicability rate among the discovered in a specific experiment will not be controlled. The implemented software in MPD offers to do that.

### Global replicability: The role of databases in the adjustment

By making use of the IMPC data to estimate the GxL-factor, we manage to stage the most realistic, but perhaps most challenging to obtain, setting for checking the GxL-adjustment in regular experimental work. The 3 tasks of (i) generating the GxL-adjustment; (ii) establishing the multi-lab “ground truth”; and (iii) estimating the performances of the adjustment in single-lab studies, are conducted each in an independent set of laboratories: by the IMPC multi-lab data, by the 3-lab experiment, and by the MPD data, respectively. Unfortunately, using IMPC data for this purpose has an inherent limitation: The standardized way by which this multi-lab study is conducted, might not reflect in full the variability among typical labs that may differ in the protocol being used, its execution, and local conditions. This interaction variation should be the one captured by an estimate of the GxL-factor, rather than the somewhat artificially smaller GxL-factor yielded by the coordinated IMPC endeavor. Thus, it is not clear whether the type I replicability error of 24% we get using the IMPC-based adjustment, which is higher than the 10% we get using the 3-lab data for adjustment (on the same single-lab results), reflects the more realistic setup or the less realistic source of data. Nevertheless, even if the actual implementation of our approach bounds the type I replicability error at somewhere between 24% and 10%, it is far more comforting a number than the 61% offered by doing nothing.

These results further indicate that extending the current effort in the MPD to utilize varied experimental results, created under no special standardization, for estimating the GxL-adjustment, should yield better results than merely relying on a single large initiative such as IMPC. Because the breadth of phenotyping in the IMPC was necessarily limited, while the breadth of archival experiments is potentially unlimited, the use and expansion of a database of research results such as MPD is a promising approach for evaluating replicability across a wide range of experiments. MPD houses thousands of well-curated physiological and behavioral phenotypic measures, and each is stored with detailed protocol information that will allow users to choose a collection of data sets that has relevant procedural, environmental, and genotypic characteristics for estimation of GxL. By coupling the GxL replicability estimator analysis tool to this database (see 4.5.4), we have enabled users a simple and convenient means of evaluating the replicability of their findings and contributing data to future users wishing to do the same. The utility of the approach grows as the breadth and depth of the data resource is expanded. Global analyses of replicability within the MPD can inform the refinement of phenotyping paradigms in many areas of research.

We note that some investigators might be hesitant to employ a test that is dependent on the scope and quality of external data. Yet, replicability is about the relation of the result of the study to results of other, possibly future, studies, and therefore cannot be self-contained in this sense. We provide an approach that allows researchers to estimate how well a single study might replicate based on prior related work. In this role, the GxL-adjusted analysis should amend, rather than replace, the usual single-lab test results and confidence intervals. One might argue about the relevance and sufficiency of the prior work, or rather conclude that the study at hand does not generalize to the conditions reflected in the prior work, as explanations for poor predicted replicability. Using another source of data for the GxL-adjustment to study the sensitivity of such prediction is feasible and may be important before attempting a costly experiment, but the ultimate proof of replicability is by conducting additional experiments.

### Translational impact: GxL-adjustment of drug treatment discoveries

A practical limitation of our effort to experimentally verify the utility of the GxL-adjustment for drug treatment experiments was the small number of results that were available for testing our approach. Indeed, there were only 4 strain differences in which the original and the adjusted analysis differed. However, relying on the analysis in Section 2.2.2, which offered hundreds of potential differences, by including many more genotypes, using the adjustment weeds out a large number of original discoveries. Of course, we have no way to verify the genotype differences that were not tested in the 3-lab experiment, so performance based on the number of non-replicable discoveries prevented and decreases in statistical power cannot be estimated, but the impact should be large. Reassuringly, the GxL-factor estimated for fluoxetine effects on many phenotypes was larger by merely 5% than the value for control mice, and the 3-way interaction was close to that value. These 2 results suggest that it may mostly be a property of the phenotype used. Future work should establish whether the interaction of Lab-by-Drug-by-Genotype does not depend critically on the drug administered.

Our results should not be used to draw conclusions about the clinical efficacy of fluoxetine, since the traditional TS test does not necessarily predict anti-depressant efficacy in humans [26], and is therefore only used here to test the replicability of previously published pharmacological results in mice, which were available for us in MPD.

### The quest for better phenotypes

It follows from our work that for an endpoint to be useful, its design should take into consideration the size of its GxL-factor *γ*. This factor compares the interaction variability to the animals’ variability, a point of view that may be different from current thinking. Body weight has low variability among animals, i.e., high precision, but its interaction term measured here was high (ages at were approximately the same but diets were not standardized). At the same time, tail suspension is notoriously known to be of high variability, but surprisingly the interaction is small (see also S4 Fig central column), and thus, the ratio turned out close to that of body weight.

More surprisingly, in the common OF test, CT had a consistently smaller factor than DT. Indeed, DT has had the largest number of comparisons changed from replicable to non-replicable due to the adjustment. Interestingly, DT as well as the CT proved highly replicable in a previous OF test by some of us, in 8 inbred strains, some of them used in the current study, across 3 laboratories, all different than the laboratories in the current study [15]. Both the interaction and the Error SD were considerably smaller relative to the measured size, with *γ*≈0.7 (see Fig 1 and error bars in Kafkafi and colleagues [15]). However, this previous study was conducted in circular, much larger arenas (≈250 cm diameter versus ≈40 cm width in the current study), while using standardized video tracking systems, and employing standardized SEE (Software for the Exploration of Exploration) analysis for robust path smoothing and segmentation [27,28]. The DT, being a measure of change in location across time, is more sensitive than CT, a measure of location, to lab-specific tracking noise that depends on the tracking technology and parameters used in each laboratory. As demonstrated by Hen and colleagues [29], an anesthetized animal “traveled” the distance of several tens of meters due to lack of proper smoothing of the location, while the time in the center of the arena was not affected at all. It should be noted that these 2 endpoints have very different interpretations in the context of behavior and as such are not interchangeable, but rather, they illustrate that different assays have different expected levels of replicability and refinement of assays with low replicability is essential.

Lipkind and colleagues demonstrated the use of multi-lab results to explicitly improve the design of the DT and CT endpoints with robust methods to achieve better replicability across laboratories [30]. Thus, while Voelkl and colleagues recently argued against using behavioral tests [12], our stand is that behavioral testing should not be dropped but rather improved, not by specifying to finer resolution how the test should be conducted, but by directed design of test hardware and software for higher replicability.

### Conclusion

In the present study across 3 laboratories, we explored altogether 152 comparisons between mouse genotypes, some treated with a drug. Of course, not all of them are expected to reflect real differences, but 53 of them did turn out to be replicable, in the sense that they were significant in Random Lab Model analysis across 3 independent labs. This indicates that, despite the criticism expressed at preclinical research using animal models, there are replicable signals worthy of exploration. Moreover, 46 of these 53 (87% power) were already discovered by the original single-lab studies. Unfortunately for the field, the criticism is correct in expressing alarm over the rate of non-replicable discoveries that comes with such a high power: along with the 46 replicable discoveries, the single lab studies also “discovered” 59 non-replicable ones.

Two solutions are generally offered for this unacceptable situation. The first one is increasing the sample size, as argued by Szucs and Ioannidis [31]. However, our work demonstrates the limitation of this recommendation for preclinical studies: It further increases the already sufficient power in the single lab, magnifying the impact of local peculiarities by making more of them statistically significant, while the within-group variance remains the same. These peculiarities will disappear relative to the much larger interaction variability that does not decrease with increasing sample size, making the additional findings non-replicable.

A second offered solution is conducting preclinical experiments across several labs, accepting only discoveries that pass the random lab analysis, as simulated by Voelkl and colleagues using data in the literature [7] and demonstrated here in our 3-lab analysis. A similar conclusion was reached by Schooler in the field of experimental psychology [32], where he advocated independent replications across laboratories before publication, and made the commitment to conduct his future work this way. However, while this multi-lab solution does work in principle, it also raises major practical difficulties for the explorative investigator: Convincing additional laboratories to participate before any findings have been published seems to be one such obstacle, and the larger budgets required are a second obstacle. In that same work, Schooler acknowledges that “it is clearly not feasible for all researchers to follow this approach in their routine work”[32], and indeed, even in his own work, this remained an ideal not too often reached. Not the least important, a third obstacle particular to preclinical animal studies is that more animals need be sacrificed before there is an indication of a replicable and important result.

This problem therefore led to 2 similar and more practical approaches in the field of preclinical animal models. Richter and colleagues suggest heterogenization of the setup in the single-lab experiment in order to capture the variability of a multi-lab study [33]. While helpful, not all the multi-lab variability was indeed captured in that study (and see also the reanalysis in Kafkafi and colleagues [14]). Simulations of multi-lab data in Voelkl and colleagues give a more promising point of view and report better success although it is not quite clear what aspects of the experiment should be heterogenized in a single-lab scenario [7].

The second approach, the GxL-adjustment method, has been shown here to be a good surrogate for such multi-lab experiments, solving almost entirely the problem of too many non-replicable discoveries. Future cooperation of scientists in the area to enrich the publicly available databases such as those reported in MPD, where GxL-factors for new phenotypes can be estimated, as well as investing efforts to design more replicability enhancing measurement tools in the sense of having lower GxL-factors will enable preclinical research to benefit from experience and results from prior animal studies.

## Materials and methods

### Databases and replicated studies

Two phenotypic databases are employed in this study: the MPD (phenome.jax.org, Bogue and colleagues [18]) and the IMPC (mousephenotypes.org [17]). MPD includes previous single-lab mouse studies submitted by data contributors, and here, we attempt to replicate across 3 laboratories some of the results in these experiments. The IMPC data was used to estimate interaction terms across several IMPC centers in Kafkafi and colleagues [14].

Results from 5 independent studies in the MPD include 4 tests that were chosen to be replicated (MPD study code names as they appear on the MPD website): Wiltshire2 [20]: Open Field (OF), Tail-Suspension (TS); Tarantino2 [21]: OF; Crabbe4 [22] Grip Strength (GS); Tordoff3 [23]: Body Weight (BW); Crowley1 [24]: BW.

Several considerations led us to select these studies: While searching the MPD for studies comparing many genotypes on many phenotypes, we nevertheless had to limit the number of animals being tested in our 3-lab experiment, by testing each mouse for several phenotypic endpoints. We therefore looked for studies in the MPD which: (i) shared the same genotypes and sexes; (ii) shared the same phenotypes; (iii) the phenotypes were also limited to those for which data from IMPC was available for the interaction terms; (iv) maximize the number of statistically significant findings in the MPD studies, since these are the only ones that might potentially be refuted by the GxL-adjustment. Still, whenever selecting several phenotypes and genotypes, many differences were not statistically significant in the original studies. The resulting design has 51 groups of mice sharing same sex, genotype, and testing lab. Seventeen of these received Fluoxetine.

**Fig 5:**
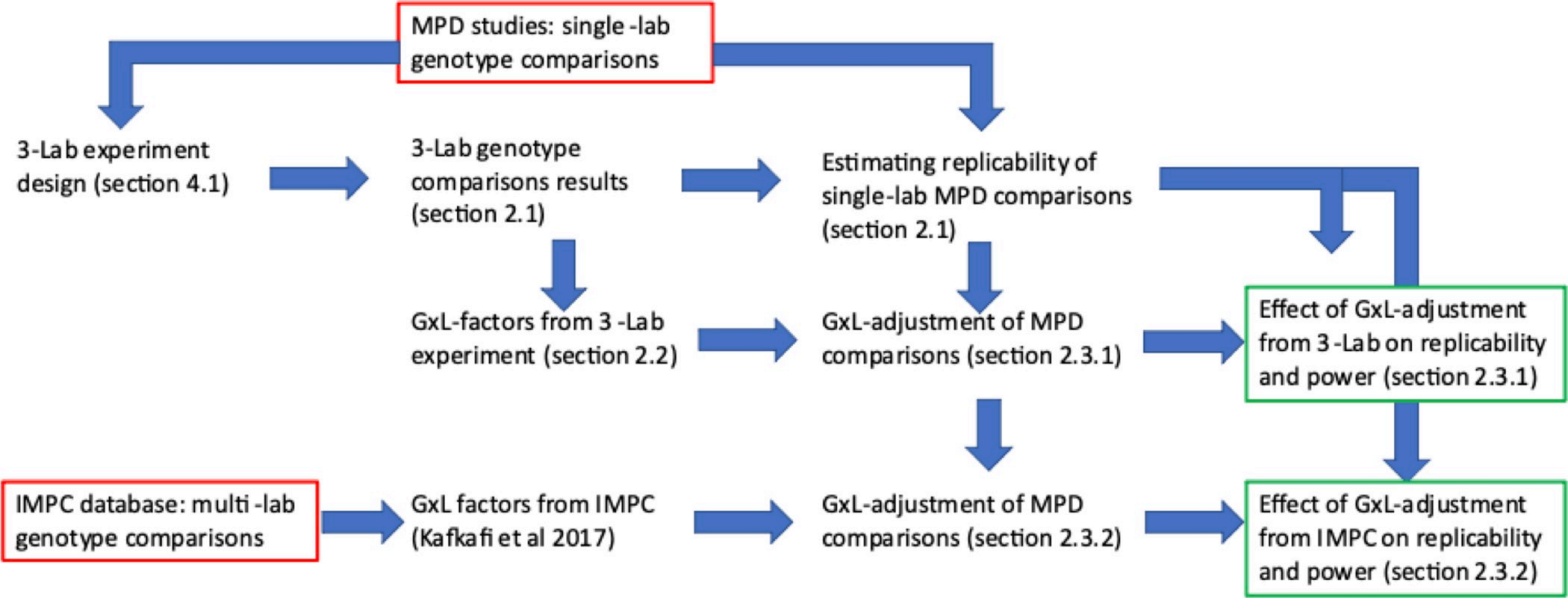
A flowchart summarizing the research process. Databases of phenotyping results appear in red frames, and final conclusions in green frames. Sections in parentheses describe the process in detail, in the Results and Methods chapters, and in references.

Following the ARRIVE 2.0 guidelines [34], the process of the research is succinctly summarized by the flowchart in Fig 5, with references to the detailed explanations in the text. The design of the 3-lab experiment, and the number of animals available per each group, are presented in S5 Table. For other details per these guidelines, see Sections 4.2–4.6 below.

### Laboratories, housing, husbandry

The 3 labs replicating the MPD studies were: The Center for Biometric Analysis (CBA) core facility The Jackson Laboratory, United States of America (JAX) under Bogue’s supervision; the laboratory in The George S. Wise Faculty of Life Sciences, Tel Aviv University, Israel (TAUL), under Gozes’ supervision; the laboratory in the Faculty of Medicine, Tel Aviv University, Israel (TAUM) under Golani’s supervision. At JAX, mice were housed in the CBA animal room and testing was conducted in CBA procedure rooms; in TAUL, mice were housed in the Faculty of Life Animal House, and test were conducted by NK in the behavioral room in this facility. In TAUM, the animals were housed in David Glasberg Tower for Medical Research, and tests were conducted by Eliezer Giladi (EG) on the 6th floor of the Tower at the Myers Neuro-Behavioral Core Facility.

Mice were housed in micro isolation cages (Thoren Duplex II Mouse Cage #11) on individually ventilated racks (THOREN Caging Systems, INC) in JAX, and in Lab Products IVC RAIR hd caging systems at TAUL and TAUM. Reverse osmosis, acidified water was provided in all, and standard autoclaved rodent diet (Purina Lab Diet 5K52) at JAX and Altromin irradiated 1318 at TAUL and TAUM ad libitum. Aspen shaving was used for bedding, humidity at 30% to 70% and temperature was set at 22°C at all 3, with ±2°C variation at TAUL and TAUM. Cage sanitation occurrs on an every 2-week basis, and no more than 5 animals per cage in all. Sex of the experimenter was mixed at JAX and male at TAUL and TAUM; 12:12-h light:dark cycle was in all 3, with lights on at 0600 h at JAX and at 0700 at TAUL and TAUM.

Note that the 2 labs in Tel Aviv University were in separate faculties and buildings, had separate experimental animals, facilities and technicians, and worked in independent time schedules. We took special care not to coordinate these 2 laboratories, as if each of them conducted the experiment in an independent study. Veterinary and Animal Care inspections in these 2 laboratories are both conducted by the TAU Center for Veterinary Care. All animal procedures in TAU were approved by TAU Institutional Animal Care and Use Committee and the Israeli Ministry of Health (protocol #04-19-061). At JAX, the work was performed under The Jackson Laboratory Animal Care and Use Committee approved protocol (#10007–1 Behavioral Phenotyping of Laboratory Mice).

### Animals and drugs

All 3 labs used the inbred strains: BALB/cJ, BTBR T^+^ Itpr3^tf^/J (BTBR), C57BL/6J, DBA/2J, SWR/J. The strain CBA/J was also used at TAUM and TAUL, but not at JAX. Breeders were transported from The Jackson Laboratory to each TAUM and TAUL and were distributed to cages of 1 male and 2 to 3 females, and 1 to 4 litters from each breeder cage were then separated at ages of approximately 50 to 60 days old, to cages of 2 to 5 mice of the same strain and sex. For the JAX experiment, mice were shipped from The Jackson Laboratory’s production facility directly to JAX CBA and mice were identified by ear punch. In TAUL, mice were transferred to smaller cages in a different room than the breeders and were identified by tail marks. Half of the male mice were administered 18 mg/kg/day fluoxetine in drinking water (see below). Males were treated with fluoxetine, while females were not, because there were no such MPD experiments in females that we could attempt to replicate. Mice were tested at similar ages (see below).

Fluoxetine HCl was purchased from Medisca, Lot 172601 (Plattsburgh, New York, USA) for the JAX experiment. In TAU, commercial fluoxetine HCl in 20 mg capsules was purchased from Ely Lilly, Israel. As in Wiltshire2 [20], the average weight measurements for each strain, together with previously determined daily water intake for each strain, were used to determine the amount of fluoxetine required to provide a daily oral dose of 0 or 18 mg/kg/day per mouse in drinking water. Male mice were treated daily with fluoxetine or water throughout the experiment. Fluoxetine treatment started at 5 to 6 weeks of age, in order to ensure 3 weeks of treatment before testing. In TAUM, the content of the water bottles changed every 3 days, while in TAUL and JAX they were changed every week, due to the use of larger bottles.

### Number of animals in the 3-lab experiment

We designed the experiment for at least *n* = 10 mice per group. In some groups, the number of animals was larger, due to litter sizes. Each group participated in more than 1 test, adding to 252 testing groups. In 52 of these, there were 9 animals. However, in tail suspension testing in JAX, some mice climbed their tails and no measurements were available, resulting in 4 measurements per group in 4 groups of females; 6 measurements per group and 8 measurements per group in 4 and 3 groups of males, respectively. In 6% of the groups, there were 15 or more animals. The number of measured animals per each tested group is given in the Supporting information S7 Table.

### Number of animals in the databases

In the previously reported MPD experiments, the number of mice per group ranged from 5 to 20, with median of 10.5 and interquartile range of 5. In the IMPC data, the number of mice per group ranged from 3 to 403, with a median of 8 and interquartile range of 3. We obviously had no control of the number of mice in MPD and IMPC.

### Tests, phenotypes, and testing parameters

This study approximately followed the IMPC behavioral tests and protocols of OF, GS, and BW, from the behavioral pipeline of the IMPC IMPReSS EUMODIC pipeline 2 https://www.mousephenotype.org/impress/PipelineInfo?id=2 [35]. In addition, we replicated the TS test in Wiltshire2 [20], which is not included in the IMPC pipelines (see Statistical methods).

The interaction terms of genotypes with the laboratory were previously estimated across multiple laboratories by Kafkafi and colleagues and were also measured in experiments submitted to MPD (see “Databases” above) [14]. Experiments and genotypes were chosen to maximize the number of tests and phenotypes with previous interaction terms from multiple labs, as explained in the considerations below. The OF test included the phenotypes (S1 Table) of DT and the percentage of time spent in the center (CT). The TS test measured the percentage of time spent immobile, and the GS test measured forepaws peak grip strength, using the average of 3 consecutive measures.

Due to the differences between the IMPC pipeline and the different MPD studies, as well as local constraints in the 3 labs, it was not feasible to precisely standardize the identity of tests, their order, and the ages in which they were conducted. Indeed, such precise standardization does not represent the realistic situation of the field and is unsuitable to the objective of this study. However, age differences at the time of each test were at most 5 weeks, and all mice were postpubertal and relatively young adult ages, i.e., not middle aged (12 month) or aged (≈18+ months). S1 Table summarizes the timelines in the databases, replicated MPD studies and the 3 labs. For similar reasons, the parameters and conditions of each test were not precisely standardized. These differences are detailed below, and for the OF test are also summarized in S2 Table.

In the OF test, the phenotypes of DT and percent of time spent in the center, in a small arena, for 10 and 20 min were recorded. In TAUL and TAUM, these were also measured in a large arena (S2 Table).

### Open field (OF) methods

Mice were allowed to acclimate to the testing room for a minimum of 60 min. Arena parameters were slightly different in the IMPC database, MPD studies, and the 3 replicated labs and are summarized in S2 Table. The apparatus was a square chamber, either 27×27 cm (“small”) for males (as in Wiltshire2) or 40×40 cm to 50×50 cm (“large”) for females (as in Tarantino2 and the IMPC protocol). In TAUL and TAUM, the males were also tested in a large arena, a week after all the other tests were concluded (S2 Table) in order to facilitate comparisons with the IMPC results.

Center and periphery definitions were also different (S2 Table). The session duration was 20 min (as in the IMPC protocol), but all analysis was done for the first 10 min (as in Wiltshire2) as well. In each test, the total DT and the percentage of session time spent in the center of the arena (CT) were measured. In addition, TAUL and TAUM also tested the control and fluoxetine males in a second OF session in the “large” arenas (as in the IMPC database) about a week after completing all other tests. Between subjects, the arena was cleaned with 70% ethanol. In TAUL, mice were tested 4 at a time in 4 square Plexiglas arenas. To begin each test, the mouse was placed in the center of the arena. The apparatus was a square chamber either 27×27 cm or 50×50 cm. Tracking and analysis was conducted using a Noldus EthoVision video tracking system. In TAUM, mice were tested 4 at a time in 4 square Plexiglas cages. To begin each test, the mouse was placed in the center of the arena. The apparatus was a square chamber either 27×27 cm or 50×50 cm. Video tracking and analysis was done with Noldus EthoVision video tracking system. In JAX, mice were acclimated to the testing room for a minimum of 60 min. The apparatus (Omnitech Electronics, Columbus, Ohio, USA) was a square chamber (40×40 cm). To begin each test, the mouse was placed in the center of the arena. Data were recorded via sensitive infrared photobeams and collected in 5-min bins.

### Grip strength (GS) methods

Three trials were carried out in succession measuring forelimb-strength only and averaged, as in the replicated MPD experiment Crabbe4 and the IMPC protocol [22]. The mouse was held by the tail, lowered over the grid, keeping the torso horizontal, and allowing only its forepaws to attach to the grid before any measurements were taken. The mouse was pulled gently back by its tail, ensuring the grip the on the top portion of the grid, with the torso remaining horizontal. The testing area was cleaned with 70% ethanol between subjects. In TAUL, TSE Systems Grip Strength Meter for mice was used with a mesh grid. In TAUM, a commercially available Ugo Basile Grip-Strength Meter was used with a wire grid, coupled with a strain gauge measuring peak force in kg. In JAX, mice were acclimated for 60 min prior to testing. A commercially available grip strength meter (Bioseb, Pinellas Park, Florida, USA) was used.

### Tail suspension (TS) methods

Mice were allowed to acclimate to the testing room for a minimum of 60 min prior to testing. They were suspended by their tails with adhesive tape to the top of Plexiglas cages. The percentage of time spent immobile was measured in 6 and in 7 min. Between subjects, the testing area was cleaned with 70% ethanol. We used the 7-min data, as did Wiltshire2 who reported dropping the first 1 min because all mice remained mobile throughout [20]. This led to not dropping the last minute in our results and should hardly affect differences. In TAUL, tracking and analysis was measured with Noldus EthoVision video tracking system. Polyethylene cylinders about 24 mm tall and 10 mm in diameter on the base of the tail were used to minimize the ability of mice to climb on their tails. Several mice that did manage to climb on their tails were discarded from analysis, as in the original Wiltshire2 experiment [20]. In TAUM, tracking and analysis was measured with Noldus EthoVision video tracking system. In JAX, standard Med-Associates (St. Albans, Vermont, USA) Tail Suspension Test chambers were used. Mice (10 to 12 weeks) were suspended by their tails with adhesive tape (VWR or Fisher, 25 mm wide) to a flat metal bar connected to a force transducer that measures transduced movement. The tape was extended the length of the mouse’s tail from 2 mm from base through 2 mm from tip, minimizing the ability of the mouse to climb its tail. A computer interfaced to the force transducer recorded the data.

### Statistical methods

#### GxL-adjustment of genotype effect in a single-lab study

The conventional *t* test for testing phenotypic difference between genotype x and genotype y, is:

$$\frac{\bar{x}-\bar{y}}{s_{p}\sqrt{\frac{1}{n_{1}}+\frac{1}{n_{2}}}},$$

where n_1_ is group size for genotype x and n_2_ for genotype y, and *s*_*p*_ is the pooled standard deviation within the groups. The number of degrees of freedom is *df* = *n*_1_+*n*_2_−2. The underlying assumptions are that both genotype groups are independent, that both have equal within group variances, and that the distribution of the phenotype is approximately Gaussian. Appropriate transformations of the original measurements such as log, logit, and cube root were used to make these assumptions more appropriate. The test can be modified if the variances are grossly unequal in the 2 groups.

*The random lab model for replicability*. When a phenotype is compared between G genotypes in L laboratories, Kafkafi and colleagues introduced the existence of Genotype by Lab interaction (GxL) as a random component the variance of which is $\sigma_{G\times L}^{2}$ [14]. The implication is that:

$$Var(\bar{x}-\bar{y})=\frac{\sigma^{2}}{n_{1}}+\frac{\sigma^{2}}{n_{2}}+2\sigma_{G\times L}^{2}.$$

This alters the *t* test as follows:

$$\frac{\bar{x}-\bar{y}}{\sqrt{s_{p}^{2}(\frac{1}{n_{1}}+\frac{1}{n_{2}})+2\sigma_{G\times L}^{2}}}.$$

*GxL-adjustment from a database*. We define the GxL-factor *γ* estimated from the results of a multi-lab study or database, as the square root of the ratio of the interaction variance to the pooled within-group error in the variance:

$$\gamma =\sqrt{\frac{\sigma_{G\times L}^{2}}{\sigma^{2}}.}$$

Note that *γ*^2^ is the environmental effect ratio (EER) of Higgins and colleagues [19].

When using GxL-adjustment at the single lab, the *t* test, with its estimated standard deviation *s*_*p*_, becomes

$$T=\frac{\bar{x}-\bar{y}}{s_{p}\sqrt{(\frac{1}{n_{1}}+\frac{1}{n_{2}})+2\gamma_{}^{2}}.}$$

The underlying assumption is that this single-lab study comes from the same population as the multi-lab study, but the local measure may have a different multiplicative scale which is reflected by the ratio of the within group standard deviations. Hence, ${{s_{p}}^{2}\gamma }_{}^{2}=\frac{{s_{p}}^{2}}{\sigma^{2}}\sigma_{G\times L}^{2}$ is an estimator of the interaction in the new study. Multiplying the database estimated interaction term by the ratio of the standard deviation in the adjusted single lab to the pooled variance in the multi-lab database translates the interaction term in one set of labs into a more relevant one in the single lab.

In a preclinical experiment involving laboratory mice and rats, a typical batch size is of 10 to 20 animals, hence, 1/*n*_*i*_ is typically smaller than 0.1. This gives us an interpretation of the size of *γ*^2^: *γ*^2^≈0.1, namely *γ*≈0.3, is of the same order as *1/n*, and *γ*^2^>1 would have a very large effect. Note that the group sizes *n*_1_, *n*_2_ do not have any effect on the GxL-factor *γ*. That is, if the interaction variance is large, increasing the number of animals can hardly improve replicability.

The distribution of T is approximated by Student’s *t* with *v* degrees of freedom using the Satterthwaite formula: Let *n*_*L*_ and *n*_*s*_ denote the number of labs, and genotypes used for estimating *γ*^2^.


$$\nu =\frac{{s_{p}^{4}(\frac{1}{n_{1}}+\frac{1}{n_{2}}+2\gamma_{}^{2})}^{2}}{{s_{p}^{4}(\frac{1}{n_{1}}+\frac{1}{n_{2}})}^{2}\frac{1}{n_{1}+n_{2}-2}+4s_{p}^{4}\gamma^{4}\frac{1}{\left(n_{L}-1)(n_{S}-1\right)}}$$

### Using GxL-adjustment for treatment effect

The database which we use for estimating the adjustment size *γ* only offers it for tests where animals were not treated, although tests for treatment effect are an important part of animal testing. Note that the adjusted test statistic T is still valid in cases where both groups are treated with similar treatment. Therefore, we also use it to test the replicability of strain effect when both groups are treated with fluoxetine, even though the adjustment component was estimated via untreated animals.

Multi-lab experiments that also included treatment groups are analyzed using a random-lab 3-way analysis, where treatment effect is added as a fixed effect, in addition to the factors we originally have in the random-lab 2-way analysis. We also include interactions involving treatment of all orders, namely, treatment-by-genotype, treatment-by-lab, genotype-by-lab, and treatment-by-genotype-by-lab where the proportional SD of the latter interaction to error SD is denoted by *γ*_*T*×*G*×*L*_.

In their paper, Wiltshire2 test the effect of fluoxetine treatment for each genotype (see figure in https://phenome.jax.org/measureset/38005) for the DS phenotype comparing treatment effects between the different strains [20]. In such a case, a linear contrast could have been performed for statistically based inference as follows:

$$\frac{\left({\bar{x}}_{T}-{\bar{x}}_{C})-({\bar{y}}_{T}-{\bar{y}}_{C}\right)}{s_{p}\sqrt{\frac{1}{n_{1T}}+\frac{1}{n_{1C}}+\frac{1}{n_{2T}}+\frac{1}{n_{2C}}}},$$

where ${\bar{x}}_{T},{\bar{x}}_{C}$ denote the mean size at the treatment and control groups of the first genotype, respectively, and *n*_1*T*_, *n*_1*C*_ denote their sample sizes. For the second genotype, ${\bar{y}}_{T},{\bar{y}}_{C}$ denote the mean size at the treatment and control groups, respectively, and *n*_2*T*_, *n*_2*C*_ denote their sample sizes.

The second-order interaction of treatment by genotype is the fixed parameter we try to estimate. All other second-order interactions, namely treatment-by-lab and the genotype-by-lab, cancel out in the contrast. What remains is the third-order interaction treatment-by-genotype-by-lab (TxGxL) that specifies the random contribution of the laboratory on the measurement for a specific genotype when treated and another one when not. Since there are 4 of these and they are independent, they increase the variance by $4s_{p}^{2}\gamma_{T\times G\times L}^{2}$,

$$\frac{\left({\bar{x}}_{T}-{\bar{x}}_{C})-({\bar{y}}_{T}-{\bar{y}}_{C}\right)}{s_{p}\sqrt{(\frac{1}{n_{1T}}+\frac{1}{n_{1C}}+\frac{1}{n_{2T}}+\frac{1}{n_{2C}})+4\gamma_{T\times G\times L}^{2}}}.$$

The degrees of freedom are recalculated again according to the Satterthwaite formula, as previously mentioned, we do not have an outer (independent) source to estimate the size of this interaction. Therefore, we estimate it using the data collected by our 3-lab experiment, and we apply it on the t-tests of the original Wiltshire2 study. Due to the lack of a third party estimate, we are unable to demonstrate the power and type I error of the tool, but merely assess the robustness of the original study results to the proposed adjustment.

A note on power analysis: Since the GxL-factor can be known prior to the design stage of an experiment, the usual formula (or software) for setting the number of animals per group can be used, all but iteratively. The standard deviation of the measured endpoints *σ* is needed as input as well as the power at a given alternative. Assuming equal group sizes the output will be *n*_1_. Set a second deviation *σ*_1_ as follows:

$$\sigma_{1}=\sqrt{\sigma^{2}+n_{1}\gamma_{}^{2}}.$$

And get *n*_2_. Repeat until the changes in *n*_*i*_ are practically small.

It is important to realize, though, that there may be a limit to the power that can be achieved because the interaction term does not diminish to 0 as the number increases, as does the first term. Thus, the study of the exact expression and its implications is left for future research.

### Displaying the results

To study the effect of the adjustment, we use our 3-lab replication mixed model analysis for establishing replicability, where a statistically significant difference is considered a “replicated difference” (Table 1, top part first column). For each difference between 2 strains in each phenotype, we then examine if (second column) this difference was significant in the original single-lab study in the MPD and if (third column) it is still significant after correcting it using the GxL-adjustment calculated in the IMPC [14]. The 6 possible combinations are denoted by categories A–F (fourth column), with their interpretations (fifth column).

We derive the proportion of the non-replicable differences (categories D+E+F) that were inappropriately discovered by the original analysis study (D+E). We also derive the same proportion after both readjusted (D). As to loss of replicable discoveries, the proportion of replicated ones discovered by the original analysis and after its adjustment are given by the last 2 lines in the table. Note that if we treat replicated difference as “true” and non-replicated difference as “false” the proportions reflect type I error and power with and without adjustment.

The reason is that if D is the number of non-replicable single lab discoveries and α* is the effective type I error of making a discovery in a single lab while it is in fact a non-replicable difference:

$$E(D)=\alpha^{*}E(D+E+F),leading\;to\;the\;above\;type\;I\;interpretation\;for\;D/(D+E+F).$$

We therefore say that the latter is an estimate of the “type I replicability error.”

Note that it might be tempting to calculate a non-replicability rate, in the same way that the false discovery rate is calculated, i.e., *(D+E)/(A+B+D+E)* or *D/(A+D)* after GxL adjustment. Alas, this ratio depends on characteristics of the original designs with the specific power for each study, and the resulting partition in our data of comparisons, which are not relevant to future experiments.

It should also be recognized that the confidence intervals given for the proportions in these tables are approximate as they are based on treating the decisions as independent.

### Computational details

Statistical analysis was done in R version 4.1.1 (2021-08-10) [36]. We use the packages “lme4” [37], “nlme,” and “multcomp” to perform multi-lab analysis via restricted maximum likelihood (REML) and pairwise comparisons [38,39]. Figures shown in this paper are produced with the package “ggplot2” [40].

## Supporting information

S1 Table Timelines of animals and tests participating in the IMPC database (EUMODIC Pipeline 2), in the 5 replicated studies in the MPD database, and in the 3 labs participating in the experiment: TAUL, TAUM, and JAX. Tests in Bold letters are common to all databases and labs.
(XLSX)

S2 Table OF parameters used in different mouse groups in each of the 3 labs, in the IMPC database and in the MPD studies. (XLSX)

S3 Table Estimated GxL-factors *γ* for different subgroups and endpoints including those estimated from 3-way analysis of drug treatment genotype and labs and those estimated from IMPC experiments. (XLSX)

S4 Table Results of naïve and GxL-adjusted genotypic differences for all phenotypes in the MPD experiments, using GxL-factors estimated from the IMPC standardized data. (XLSX)

S5 Table The design of the 3-lab experiment, and the number of available mice per group in each lab: JAX, TAUL, and TAUM. (XLSX)

S6 Table The 53 comparisons found significant in the 3-lab experiment (Table 1). (XLSX)

S7 Table The number of measured animals per each tested group in each lab (JAX, TAUL, and TAUM) and measure. (XLSX)

S1 Fig Forepaw peak strength results in the grip strength (GS) test, in the 3-lab experiment, and the MPD study Crabbe4, using boxplots (top) and genotype means after raising to the power of 1/3 transformation (bottom), in females (left), males (center), and fluoxetine-treated males (right). Black error bars represent the interaction SD and the within-group error SD. The data and R code underlying this figure can be found in https://doi.org/10.5281/zenodo.7672211.
(TIFF)

S2 Fig Distance Traveled (DT) in the Open Field (OF) test, in a small arena in 10 min, in the 3-lab experiment and the MPD study Crabbe4, using boxplots (top) and genotype means (bottom), in males (left) and fluoxetine-treated males (right). Black error bars represent the interaction SD and the within-group error SD. The data and R code underlying this figure can be found in https://doi.org/10.5281/zenodo.7672211.
(TIFF)

S3 Fig Center Time % in the open field (OF) test, in a large arena for 10 min, in the 3-lab experiment and the MPD study Tarantino2, using boxplots (top) and genotype means after logit transformation (bottom) in the 3 laboratories, in females (left), males (center), and fluoxetine-treated males (right). Black error bars represent the interaction SD and the within-group error SD. The data and R code underlying this figure can be found in https://doi.org/10.5281/zenodo.7672211.
(TIFF)

S4 Fig The % time spent in immobility during 7 min in the tail suspension (TS) test, in the 3-lab experiment, and the MPD study Wiltshire2, using boxplots (top) and genotype means after logit transformation (bottom), in females (left), males (center), and fluoxetine-treated males (right). Black error bars represent the interaction SD and the within-group error SD. The data and R code underlying this figure can be found in https://doi.org/10.5281/zenodo.7672211.
(TIFF)

S5 Fig The % center time % (CT) results in the open field (OF) test, in a large arena for 10 min, in the 3-lab experiment and in the MPD study Wiltshire2, using boxplots (top) and genotype means after logit transformation (bottom), in females (left), males (center), and fluoxetine-treated males (right). Black error bars represent the interaction SD and the within-group error SD. The data and R code underlying this figure can be found in https://doi.org/10.5281/zenodo.7672211.
(TIFF)

## Abbreviations

ADNP: activity-dependent neuroprotective protein
BW: body weight
CT: center time
DT: distance traveled
EER: environmental effect ratio
GS: grip strength
IMPC: International Mouse Phenotyping Consortium
MPD: Mouse Phenome Database
REML: restricted maximum likelihood
TS: tail suspension
