# Supplementary figures and images for: A multi-lab experimental assessment reveals that replicability can be improved by using empirical estimates of genotype-by-lab interaction
Source: PLoS Biol. 2023 May 1;21(5):e3002082. doi: 10.1371/journal.pbio.3002082 (PMC10174519; doi:10.1371/journal.pbio.3002082)

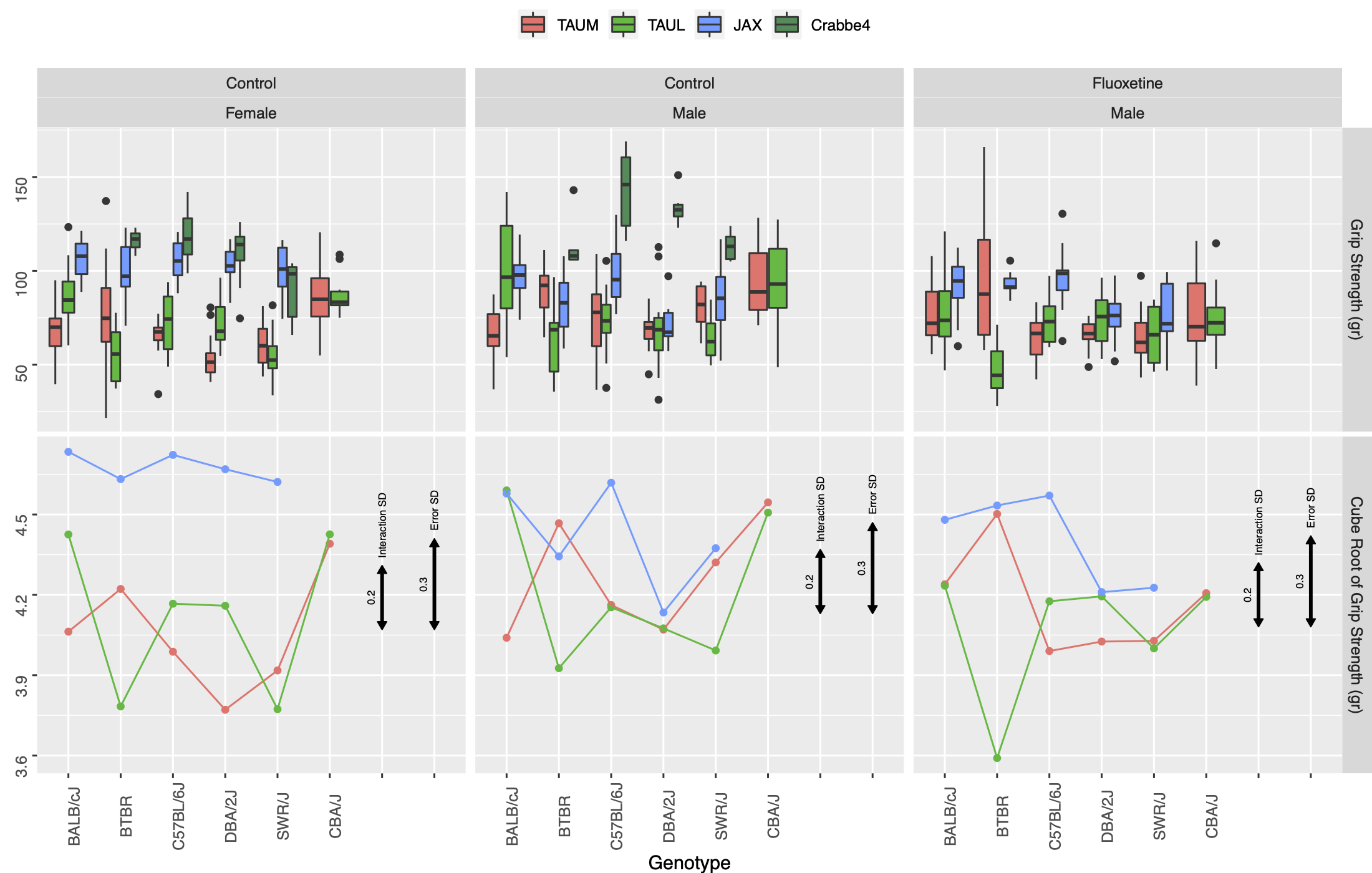

Supplement: S1 Fig — Forepaw peak strength results in the grip strength (GS) test, in the 3-lab experiment, and the MPD study Crabbe4, using boxplots (top) and genotype means after raising to the power of 1/3 transformation (bottom), in females (left), males (center), and fluoxetine-treated males (right). Black error bars represent the interaction SD and the within-group error SD. The data and R code underlying this figure can be found in https://doi.org/10.5281/zenodo.7672211. (TIFF) [file pbio.3002082.s008.tiff]

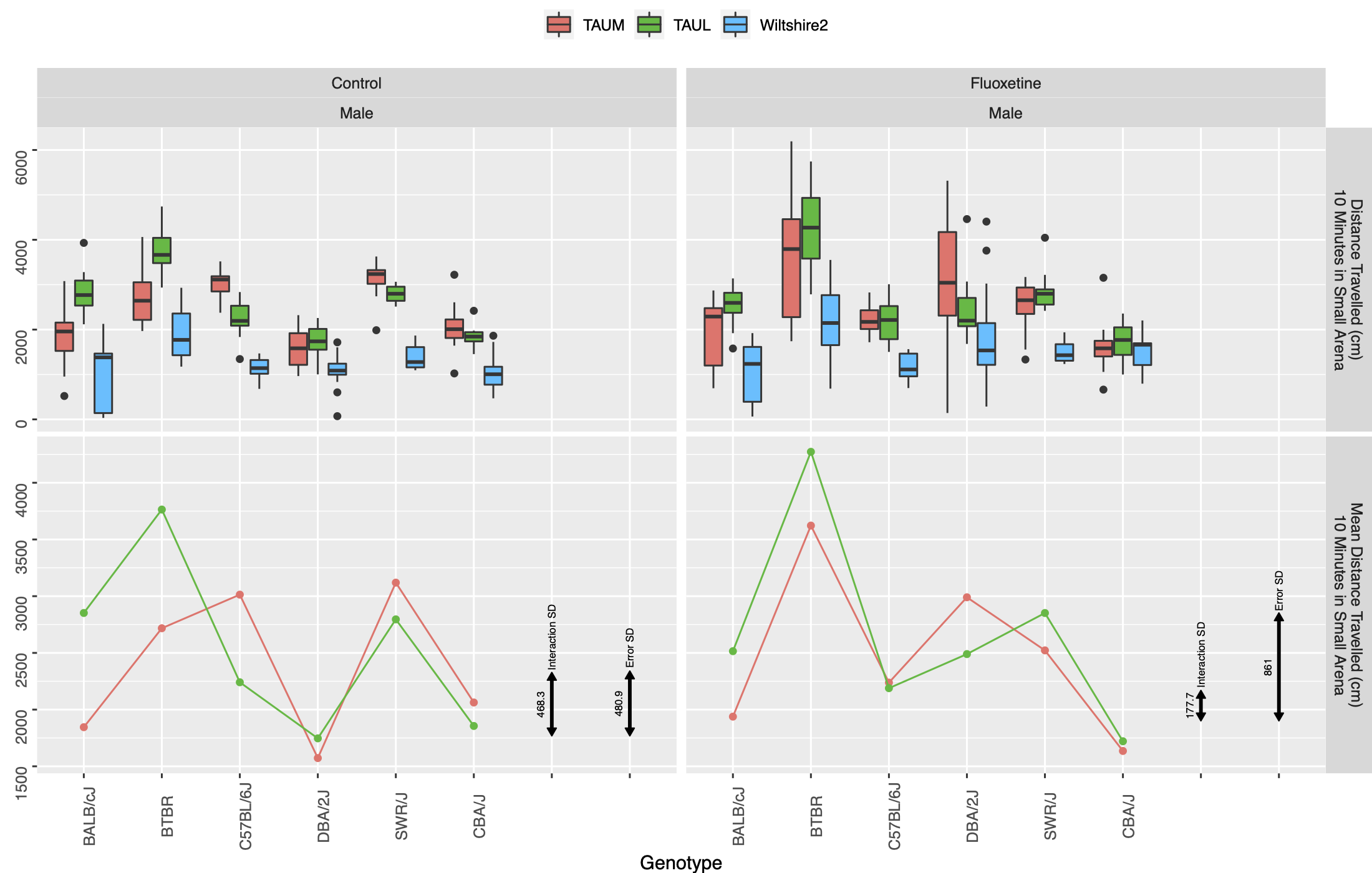

Supplement: S2 Fig — Distance Traveled (DT) in the Open Field (OF) test, in a small arena in 10 min, in the 3-lab experiment and the MPD study Crabbe4, using boxplots (top) and genotype means (bottom), in males (left) and fluoxetine-treated males (right). Black error bars represent the interaction SD and the within-group error SD. The data and R code underlying this figure can be found in https://doi.org/10.5281/zenodo.7672211. (TIFF) [file pbio.3002082.s009.tiff]

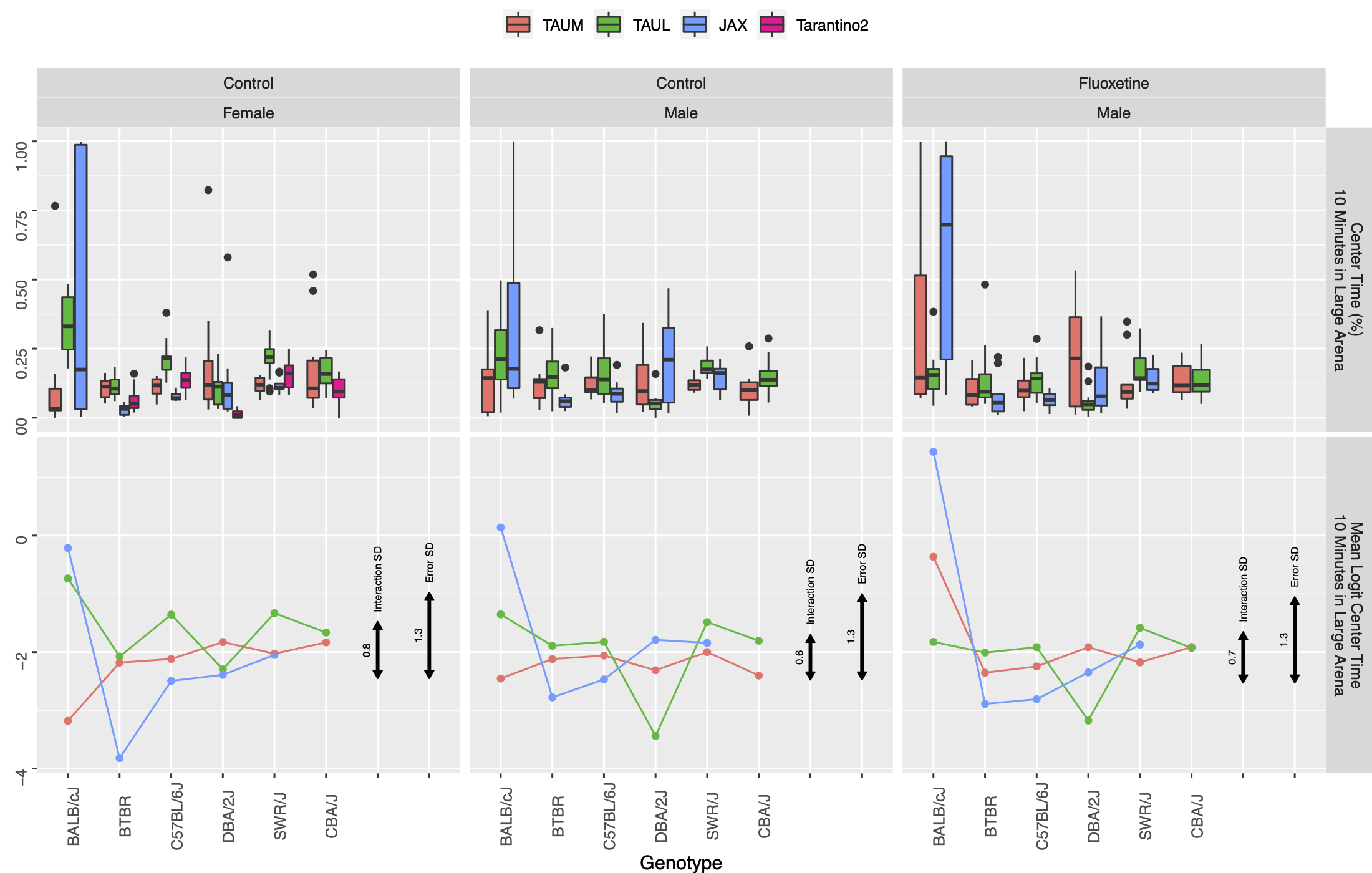

Supplement: S3 Fig — Center Time % in the open field (OF) test, in a large arena for 10 min, in the 3-lab experiment and the MPD study Tarantino2, using boxplots (top) and genotype means after logit transformation (bottom) in the 3 laboratories, in females (left), males (center), and fluoxetine-treated males (right). Black error bars represent the interaction SD and the within-group error SD. The data and R code underlying this figure can be found in https://doi.org/10.5281/zenodo.7672211. (TIFF) [file pbio.3002082.s010.tiff]

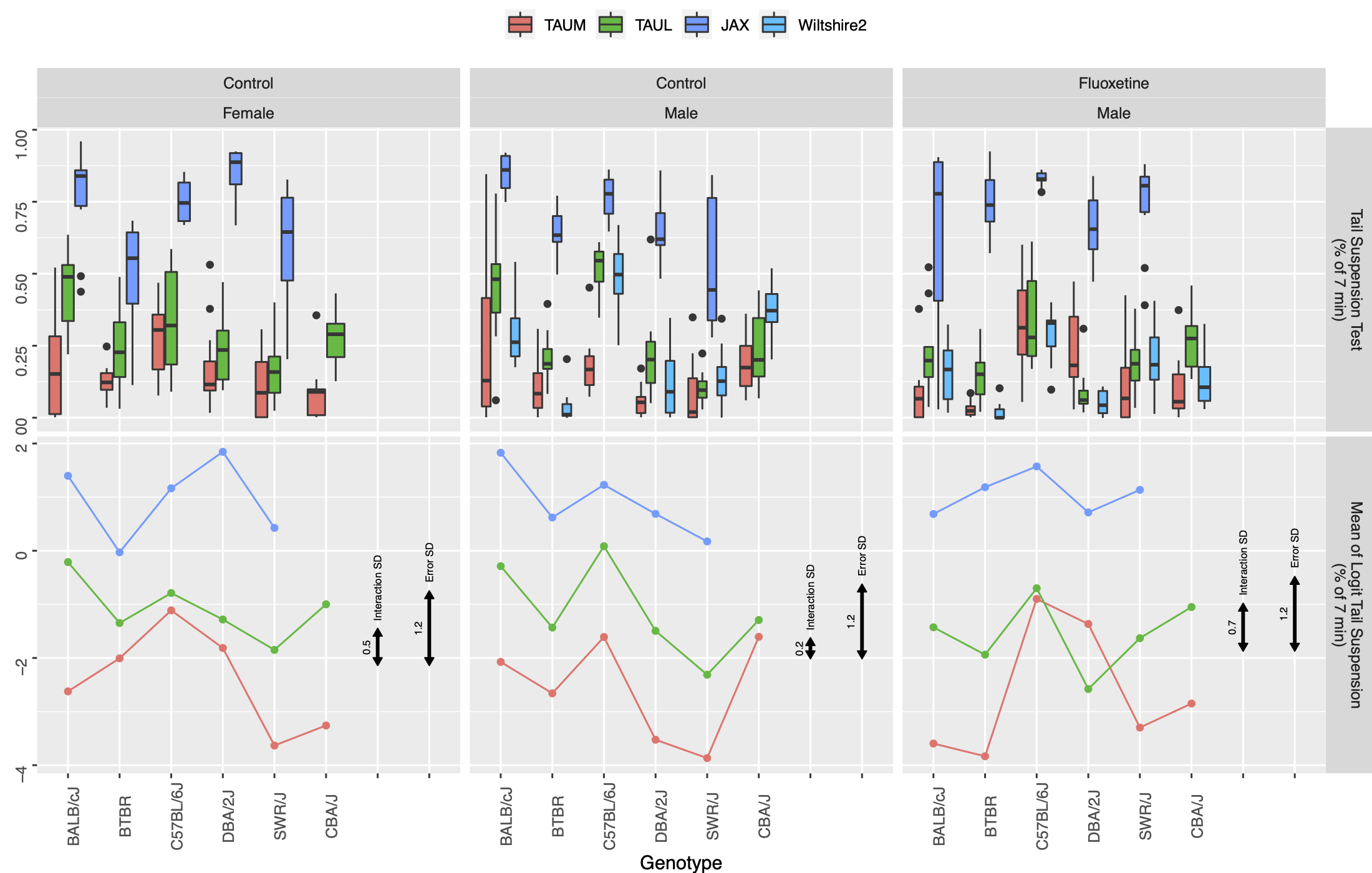

Supplement: S4 Fig — The % time spent in immobility during 7 min in the tail suspension (TS) test, in the 3-lab experiment, and the MPD study Wiltshire2, using boxplots (top) and genotype means after logit transformation (bottom), in females (left), males (center), and fluoxetine-treated males (right). Black error bars represent the interaction SD and the within-group error SD. The data and R code underlying this figure can be found in https://doi.org/10.5281/zenodo.7672211. (TIFF) [file pbio.3002082.s011.tiff]

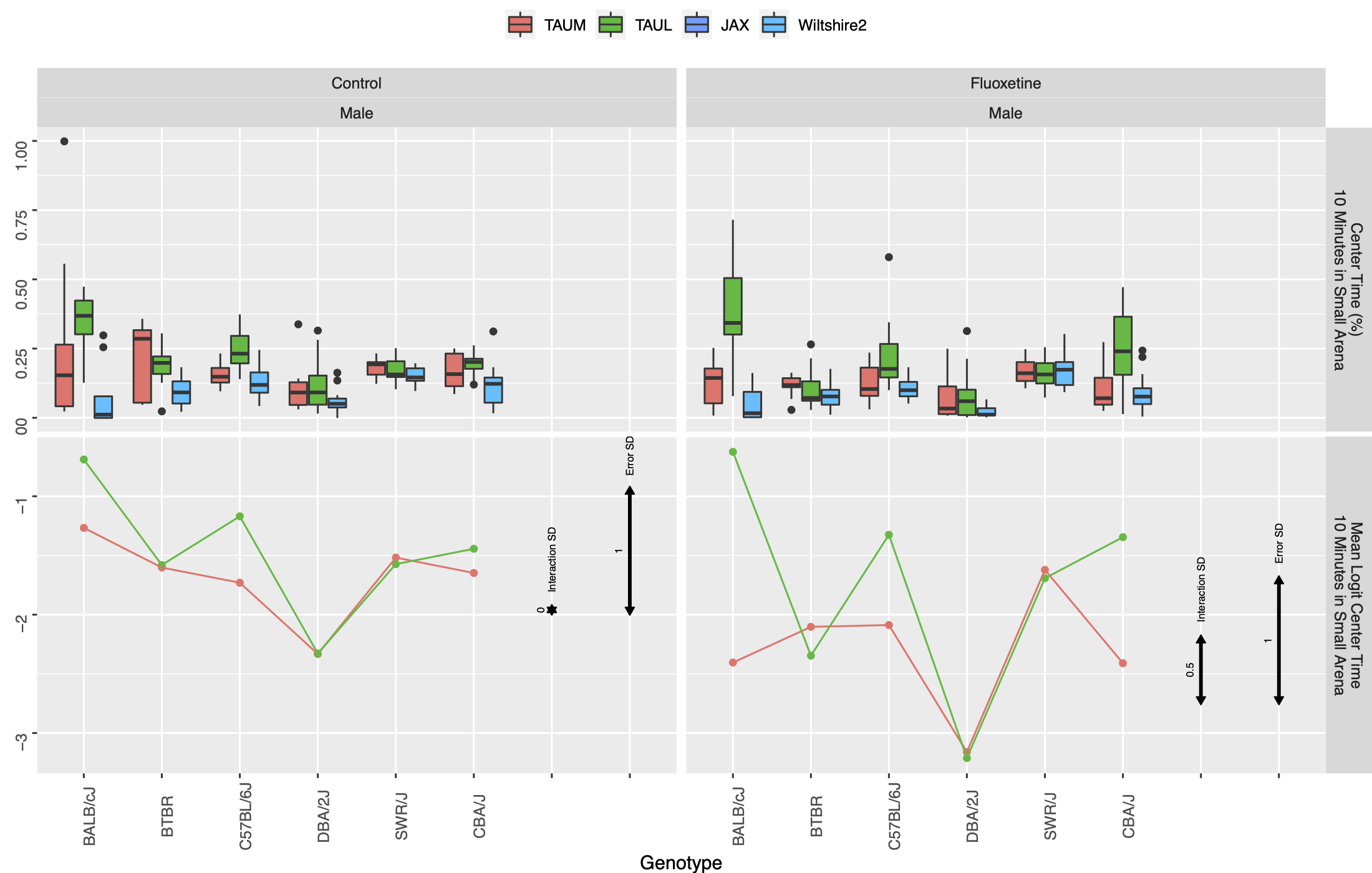

Supplement: S5 Fig — The % center time % (CT) results in the open field (OF) test, in a large arena for 10 min, in the 3-lab experiment and in the MPD study Wiltshire2, using boxplots (top) and genotype means after logit transformation (bottom), in females (left), males (center), and fluoxetine-treated males (right). Black error bars represent the interaction SD and the within-group error SD. The data and R code underlying this figure can be found in https://doi.org/10.5281/zenodo.7672211. (TIFF) [file pbio.3002082.s012.tiff]
